# Medicine–food homologous bioactives in metabolic dysregulation-associated osteoporosis: a review of preclinical evidence and potential liver–bone and gut–bone actions

**DOI:** 10.1186/s13020-026-01453-6

**Published:** 2026-07-22

**Authors:** Wen Fan, Beilei Shi, Ze Gao, Yan Cui, Xiaofeng Zhu

**Affiliations:** 1 School of Traditional Chinese Medicine, Jinan University, Guangzhou, 510630 Guangdong People’s Republic of China; 2 The First Affiliated Hospital of Jinan University, Guangzhou, 510630 Guangdong People’s Republic of China

**Keywords:** Indirect pharmacology, MFH bioactives, Osteoporosis, Liver-bone axis, Gut-bone axis

## Abstract

**Background:**

Osteoporosis is increasingly linked to metabolic dysregulation. Medicine–food homologous (MFH) materials contain diverse natural bioactives with reported osteoprotective effects, but their overall evidence landscape remains insufficiently integrated.

**Purpose:**

To synthesize preclinical evidence on MFH-derived bioactives that improve osteoporosis-related phenotypes and to discuss their possible trans-organ actions under metabolic disturbance.

**Methods:**

PubMed, Web of Science, Scopus, and CNKI were searched from January 2000 to September 2025. Original in vivo or in vitro studies were included when a defined MFH-derived constituent or standardized single-material extract was tested in an osteoporosis-relevant model and reported both bone-related and metabolism-related outcomes.

**Results:**

A total of 38 bioactive components from 24 MFH materials were identified and regrouped into seven higher-level natural-product categories, including flavonoids, phenolic and polyphenolic compounds, polysaccharides, saponins, terpenoids, proteins/peptides, and other specialized metabolites. Across studies, osteoprotective effects were frequently accompanied by parallel improvements in lipid metabolism, inflammatory status, oxidative stress, gut microbiota, or related metabolites. These findings suggest that MFH bioactives may act beyond bone-local signaling alone. In the discussion, this pattern was further interpreted through liver-bone and gut-bone.

**Conclusion:**

MFH-derived bioactives show potential to improve osteoporosis-related phenotypes and may exert broader systemic regulatory effects, although specific mediators and causal links still require validation.

**Graphical Abstract:**

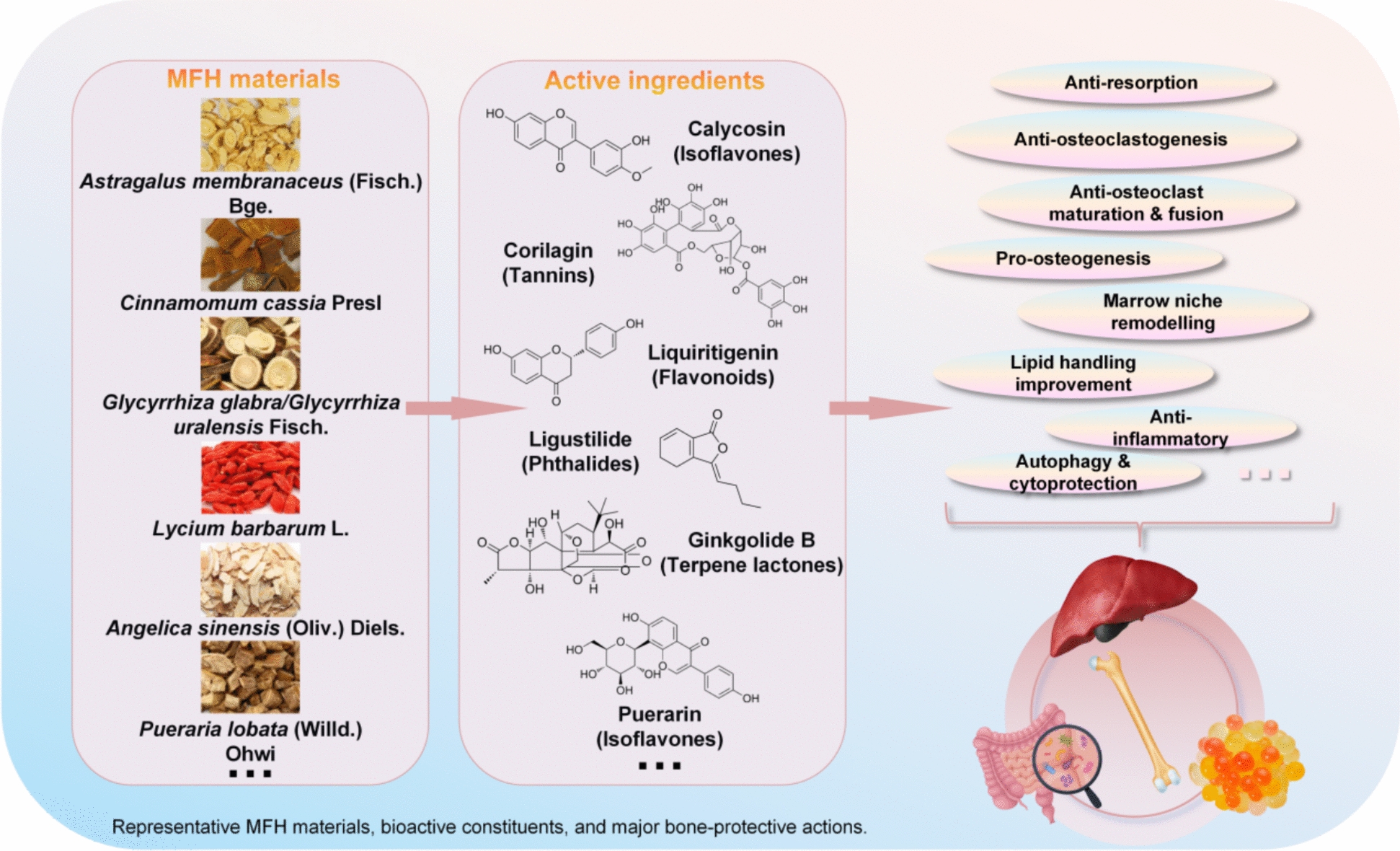

**Supplementary Information:**

The online version contains supplementary material available at 10.1186/s13020-026-01453-6.

## Introduction

Osteoporosis can be classified into two major categories based on etiology: primary osteoporosis and secondary osteoporosis. The former mainly encompasses post-menopausal osteoporosis associated with estrogen deficiency and senile osteoporosis related to aging. The latter is commonly observed in the context of long-term glucocorticoid use, type 2 diabetes, chronic inflammatory diseases, and tumor treatment. Its pathological basis often involves a combination of systemic metabolic disorders and imbalances in bone remodeling [117]. This disease is characterized by the destruction of bone microarchitecture, decreased bone density, and an elevated risk of fractures [33]. As bone mass gradually declines, patients face a significantly increased risk of fractures in areas such as the hip, vertebrae, and distal radius, which severely impairs their mobility and quality of life and imposes a substantial socioeconomic burden [38, 111]. Current prevention and treatment strategies encompass calcium and vitamin D supplementation, weight-bearing exercises, and anti-resorptive or bone-forming medications. However, long-term treatment is still constrained by issues related to patient compliance and safety, indicating an urgent need for intervention approaches that are safe, suitable for long-term use, and target multiple pathways [160]. Against this backdrop, bioactive components derived from medicinal plants, particularly those from medicine-food homologous (MFH) materials with long-term dietary applicability, are increasingly regarded as promising alternative or adjunctive strategies for osteoporosis, especially in phenotypes accompanied by metabolic imbalance.

The concept of medication-food homology (MFH) reflects the long-term practice of traditional Chinese medicine (TCM), and the development and utilization of related resources are becoming increasingly important in the context of the world's aging population. In China, 106 MFHs have been approved by official documents, which provides an operating scope for evidence synthesis and translation discussion [35]. In parallel, increasing attention has been directed to the skeleton as an endocrine organ that participates in systemic metabolic regulation. Beyond its structural role, bone-derived factors and marrow niche signals have been proposed to interact with whole-body energy and mineral homeostasis, thereby linking metabolic disturbances to bone remodelling outcomes. Although many studies have examined the roles of individual MFH components in bone metabolism, such as bone-fat axis, bone-pancreas axis, and bone-gut axis, which participate in the fine regulation of energy metabolism and bone remodeling [58, 59, 178]. This network involves key nodes such as AMPK/SIRT/PGC-1α axis [53] and Nrf2-mediated redox homeostasis [115], NLRP3/NF-κB inflammatory pathway [169], and lipid metabolism-related pathways such as PPAR/LXR [169]. There is still a lack of systematic reviews integrating its multi-component and multi-pathway evidence and explaining its overall value in metabolism-bone interactions.

Recently, indirect pharmacology has been proposed as a new perspective to understand how active ingredients exert therapeutic effects beyond the primary lesion site through cross-organ modulation. In the context of osteoporosis with metabolic disorders, this view provides an explanatory model for considering how MFH-derived biological activity affects bone remodeling not only through local bone pathways but also through broader systemic changes. Therefore, this article systematically summarizes the preclinical evidence on MFH-derived biological activities to improve osteoporosis-related phenotypes and metabolic-related abnormalities, and classifies these compounds according to their chemical characteristics. On this basis, the possible cross-organ actions of MFH bioactives under conditions of metabolic disturbance are further discussed through liver-bone and gut-bone perspectives. This paper aims to provide an integrated summary of evidence and a biological perspective for future development and mechanistic studies of bone health products based on MFH resources.

## Methods

### Databases and search strategies

**Fig. 1:**
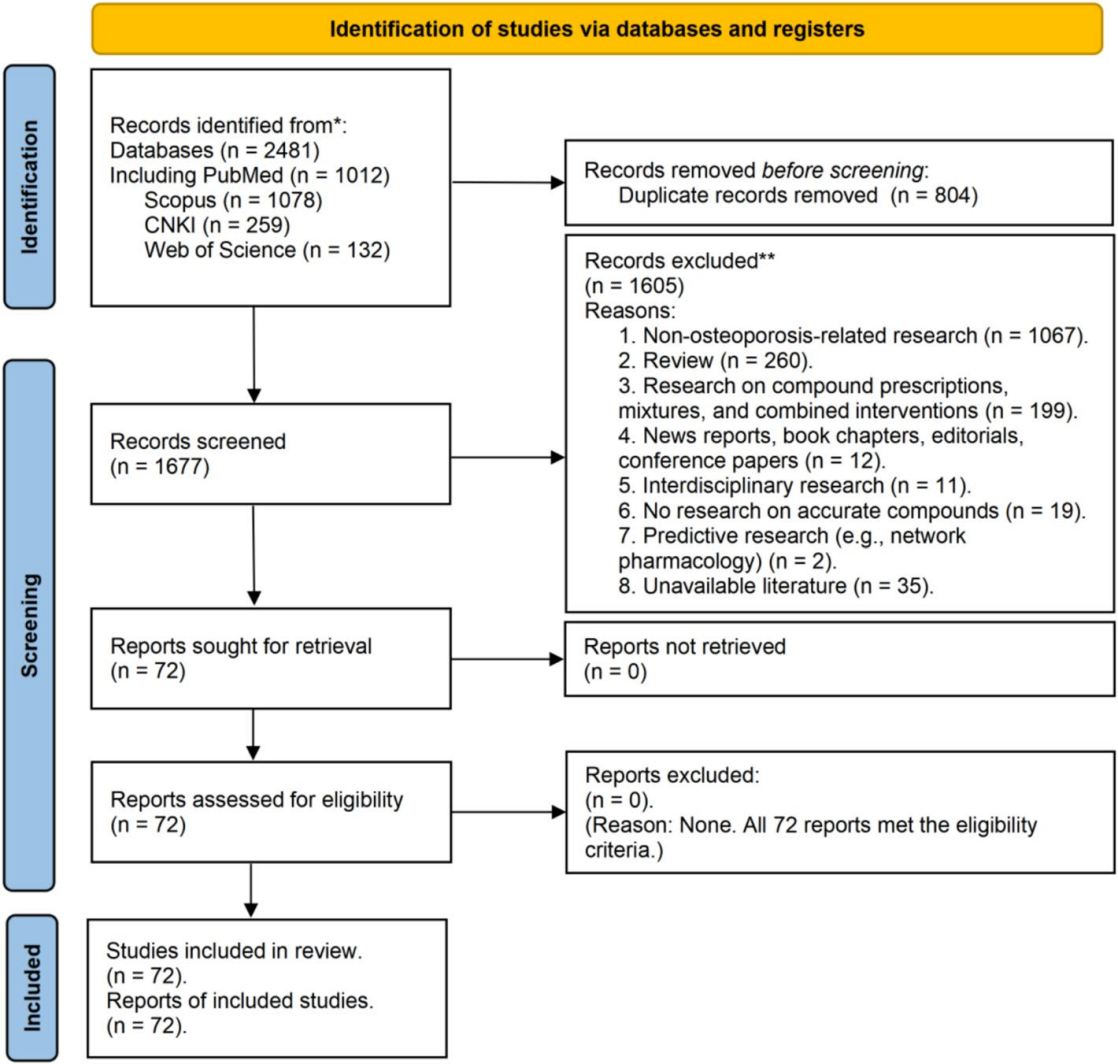
Flow chart for this review

A systematic and comprehensive literature search was conducted in four electronic databases, including Scopus, PubMed, CNKI, and Web of Science, for the period from January 2000 to September 2025. The search strategy combines subject words and free words, Keywords included "metabolism", "osteoporosis", "osteoblast", "osteoclast", and the standard Latin name of the MFH materials. The literature search used Boolean logic operators (AND, OR) to construct a combined expression. To ensure comprehensive coverage, relevant studies were initially screened by two independent reviewers, and duplicate and irrelevant studies were excluded according to the title and abstract. The candidate articles were then read in full and screened according to prespecified inclusion and exclusion criteria. Disagreements during the review process were resolved by the third reviewer through consultation. For studies in which the full text is not publicly available, reviewers will make every effort to contact the corresponding author by email to obtain the original text (Fig. 1).

### Eligibility criteria

In the present review, the term MFH material is used as a convenient shorthand for these catalogue-listed materials as a whole, with non-plant MFH ingredients (for example, poultry-derived collagen) explicitly indicated where relevant.

Inclusion criteria: (1) Intervention: the intervention was based on MFH materials or their derivatives, using a clearly defined single active constituent or a standardised extract of a single MFH material as the main intervention. Standardised extracts prepared from a single MFH material were considered eligible. (2) Outcomes: the study reported at least concurrently (i) one or more indicators of metabolic status and (ii) one or more bone-related indicators. (3) Study type: original experimental research, including animal studies and cell-based studies. (4) Availability: full-text articles available in Chinese or English, with sufficiently detailed descriptions of study design and experimental methods.

Exclusion criteria: (1) Article type: reviews, commentaries, conference abstracts, book chapters, patents, unpublished work, or purely theoretical/predictive studies (such as network pharmacology or molecular docking without experimental validation). (2) Unclear intervention: the test substance was not derived from MFH materials, or the botanical source, Latin binomial name or authentication information was not clearly stated.

### Study selection and data extraction

For all included studies, the following information was extracted: first author, year of publication, plant species, bioactive compound and its chemical class, experimental model, type of osteoporosis, dose of the extract or compound, treatment duration, and the key metabolic parameters and bone-related endpoints assessed.

## Results

### MFH material constituents with metabolic and anti-osteoporotic activities

**Fig. 2:**
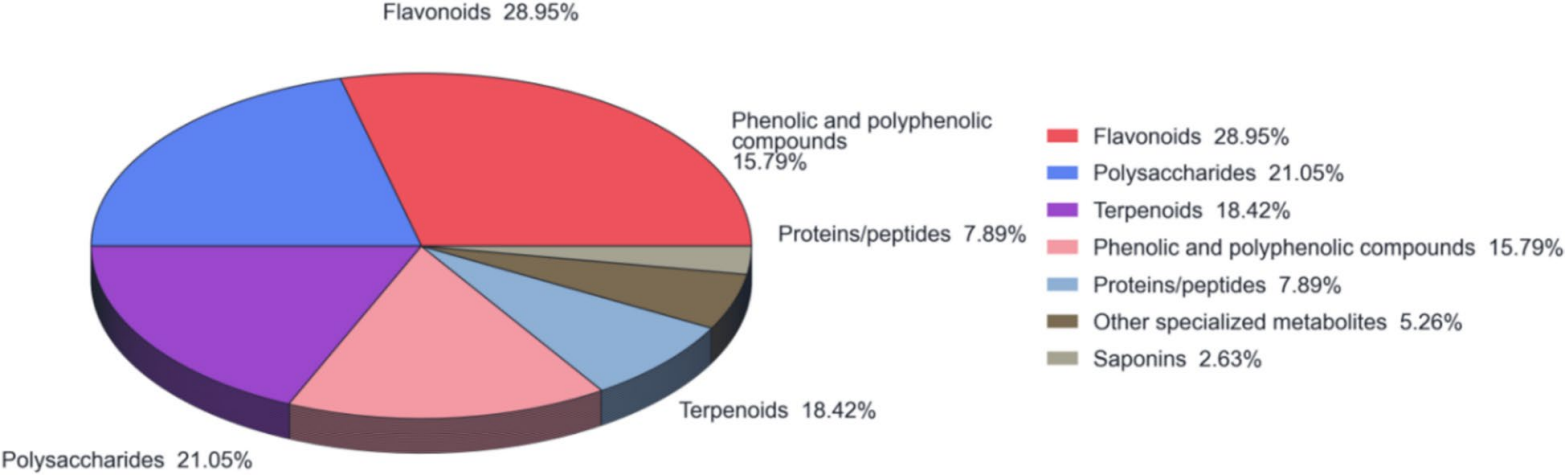
Distribution of MFH-derived bioactive constituent records across seven higher-level natural-product categories

The capacity of MFH-derived constituents to modulate osteoporosis and associated metabolic disturbances is closely related to their underlying chemical architecture. Our literature search identified 38 bioactive constituents from 24 MFH materials. To avoid overlap between parent and sub-class chemical terms, these constituents were regrouped into seven higher-level natural-product categories according to their core scaffold and biosynthetic origin: flavonoids, phenolic and polyphenolic compounds, polysaccharides, saponins, terpenoids, proteins/peptides, and other specialized metabolites (Fig. 2, Table S1). In this revised classification, isoflavones and isoflavone glycosides were included under flavonoids rather than counted as independent parallel categories. Overall, flavonoids and polysaccharides represented the most frequently reported categories, followed by terpenoids, phenolic and polyphenolic compounds, proteins/peptides, saponins, and other specialized metabolites. Across the included studies, improvements in bone phenotypes were often accompanied by changes in lipid-related abnormalities, inflammatory or oxidative status, gut microbiota composition, and short-chain fatty acid production. These co-reported changes suggest that MFH-derived bioactives may influence osteoporosis-related outcomes not only through bone-local pathways but also through broader metabolic and gut-derived regulatory signals.

### Chemical classification of MFH-derived compounds

To facilitate comparison of different chemical families and their roles in regulation of the osteoporosis with metabolic abnormalities, the compounds identified in this review were regrouped, on the basis of core scaffold and biosynthetic origin, into seven higher-level categories. Molecules bearing phenolic hydroxyl substituted aromatic rings were classified as phenolic and polyphenolic compounds. Structures with the typical C6-C3-C6 flavonoid backbone were assigned to flavonoids. Compounds with polysaccharide chains as the main structural framework, such as Astragalus polysaccharides, *Lycium barbarum* polysaccharides and *Polygonatum* polysaccharides, were grouped as polysaccharides. Steroidal or triterpenoid aglycones conjugated to sugar residues (for example astragalosides) were classified as saponins. Terpenoid molecules and their derivatives derived from the isoprenoid pathway, including iridoid glycosides, monoterpene aldehydes, phthalides, sesquiterpene alcohols, terpene lactones and triterpenes (such as total iridoid glycosides of *Cornus officinalis*, safranal, ginkgolide B and 18β-glycyrrhetinic acid), were grouped as terpenoids. Macromolecules and polypeptides composed of amino acids, including collagens and soybean derived proteins, were assigned to proteins and peptides. Low-frequency chemotypes with more distinct structural features were combined into a residual class termed other specialized metabolites.

### Flavonoids and isoflavones

#### *Astragalus membranaceus* (Fisch.) Bge

*Astragalus membranaceus* (Fisch.) Bge. is a root drug with more than two millennia of medicinal use in China, typically administered after slicing or honey-processing, and is also recognised as a MFH material [71, 113]. Among its constituents, the isoflavone calycosin has been shown to improve BMD and trabecular microarchitecture, increase serum ALP and LC3II levels, and enhance the tissue expression of Runx2 and Beclin-1. These findings indicate that calycosin not only prevents deterioration of bone microstructure but also helps to maintain bone metabolic homeostasis by promoting autophagy in bone marrow mesenchymal stem cells (BMSCs) and driving their differentiation toward the osteoblastic lineage [142]. On the metabolic side, calycosin-7-O-β-D-glucoside attenuates palmitate-induced lipid accumulation in hepatocytes through activation of AMPK [149]. It also activates Nrf2/HO-1 antioxidant axis and upregulates several downstream antioxidant enzymes, including NAD(P)H quinone dehydrogenase 1 (NQO1), glutamate-cysteine ligase catalytic and modifier subunits (GCLC and GCLM) and thioredoxin reductase 1 (TrxR1) and thioredoxin 1 (Trx1). In parallel, calycosin has been reported to modulate NF-κB signalling in spinal cord neurons, which may contribute to the dampening of neuroinflammation and, indirectly, to reduced bone resorption under conditions of metabolic stress [101]. Taken together, these data point to calycosin and its glycosides as promising *Astragalus*-derived candidates that couple osteogenic and pro-autophagic effects in BMSCs with systemic antioxidant and anti-inflammatory actions. Nevertheless, the current evidence is largely confined to cellular work and a limited number of animal models, and more detailed mechanistic and translational studies are needed to define their precise targets and therapeutic potential in metabolic osteoporosis.

#### *Glycine max* (L.) Merr.

*Glycine max* (L.) Merr. is a typical MFH material whose seeds are rich in structurally diverse isoflavones, including daidzein, daidzin, glycitin and genistein. These phytoestrogenic compounds are particularly notable for their effects on bone metabolic homeostasis and metabolic diseases.

In estrogen deficiency induced PMOP models, daidzein modulates the immune-inflammatory network and suppresses osteoclast activity. It reduces TNF-α positive CD4⁺CD28⁻ senescent T cells and ROS, inhibits osteoclast formation and restores the expression of CD28 and nucleolin [122]. Its glycoside form, daidzin, acts on both high bone turnover and lipid metabolism. Oral administration at 50 mg/kg per day significantly increases femoral BMD and bone strength, lowers urinary excretion of pyridinoline and deoxypyridinoline, and at the same time attenuates body weight gain, reduces abdominal fat accumulation and prevents ovariectomy induced elevations in serum cholesterol and triglycerides, indicating dual protection of bone and lipid metabolism. Glycitin shows a similar profile to daidzin, stabilising bone turnover, improving BMD and effectively blocking hypercholesterolaemia and hypertriglyceridaemia.

Genistein is a more extensively studied isoflavone that targets both bone and metabolic pathways. Appropriate dosing nearly restores femoral BMD to normal levels and increases osteocalcin expression. Gene expression analyses reveal that genistein upregulates osteogenic factors such as estrogen receptor alpha (ERα), transforming growth factor-β receptor 2 (TGF-βR2), fibroblast growth factor receptor 3 (FGFR3) and bone morphogenetic protein 4 (BMP4), while downregulating pro-inflammatory and matrix degrading mediators including IL-1β, IL-6, TNF superfamily member 10 (TNFSF10) and matrix metalloproteinase-13 (MMP-13), thereby exerting a coordinated effect that promotes bone formation and suppresses bone resorption [91]. In orchiectomized male mice, subcutaneous administration of genistein at 0.4–0.8 mg/d for 3 weeks significantly prevented the loss of whole-femur and distal cancellous bone BMD, restored trabecular bone volume fraction and number, and reduced trabecular separation. Its bone-protective effect was comparable to that of 17β-estradiol, without affecting seminal vesicle weight, suggesting a certain degree of bone tissue selectivity. Mechanistically, genistein corrected the orchiectomy-induced abnormal expansion of B220⁺ pre-B cells in the bone marrow, thereby suppressing excessive B-lymphopoiesis and mitigating androgen deficiency–associated bone resorption. In addition, the rate of body-weight gain in orchiectomized mice treated with genistein or estradiol was reduced, which the investigators attributed to their shared ability to modulate lipid metabolism and slow fat accumulation [41].

Despite the consistent improvements in BMD, trabecular architecture and lipid profiles observed with daidzein, daidzin, glycitin and genistein in OVX and diet-induced models, marked heterogeneity in isoflavone species, dosing regimens and outcome measures, together with scarce and partly inconsistent clinical data, means that the optimal composition, exposure window and safety profile of soybean isoflavones for metabolic osteoporosis remain to be defined.

#### *Glycyrrhiza glabra* and *Glycyrrhiza uralensis* Fisch

*Glycyrrhiza glabra* and *G. uralensis* are among the most frequently used herbs in TCM [44]. After honey-processing, their roots are widely consumed as natural sweeteners and flavouring agents in foods such as confectionery, tobacco and beer, with a sweetness estimated to be about fifty times that of sucrose [19, 45, 88, 100]. Beyond its culinary value, licorice has attracted considerable pharmacological interest. A series of in vitro and in vivo studies indicate that its representative flavonoid liquiritigenin (LTG) exerts anabolic actions on bone tissue. Mechanistically, recent work in OVX mice and MC3T3 E1 osteoprogenitors has further linked LTG’s osteoanabolic activity to restoration of autophagy lysosome proteostasis. LTG enhanced autophagic flux and auto lysosomal degradation, accompanied by increased expression of Beclin 1 and LAMP1 and reduced accumulation of the autophagy substrate p62, together with improved lysosomal acidification. In parallel, LTG suppressed mitochondria associated apoptosis by shifting the Bcl 2 Bax axis toward a pro survival state and reducing caspase 3 activation. Functionally, these coupled actions rescued both early osteogenic commitment, reflected by ALP activity, and late stage mineralisation, even under lysosomal blockade by chloroquine or apoptosis inducing stress triggered by TNF α plus SM 164, providing a coherent cellular basis for its protection against oestrogen deficiency related trabecular bone loss [98]. LTG decreases pro-inflammatory mediators and induces modest expression of HO-1 in osteoclasts, thereby attenuating the pro-osteoclastogenic influence of an inflammatory microenvironment [124]. Beyond these local skeletal and systemic metabolic effects, pharmacological studies show that LTG behaves as a partial agonist with selectivity for oestrogen receptor β (ERβ) [80]. Given that ERβ is implicated in promoting osteoblast differentiation and restraining osteoclast activity, this receptor profile provides a mechanistic rationale for the potential benefit of LTG in oestrogen-deficiency-related osteoporosis. As a comparative reference within the same flavonoid family, isoliquiritigenin from licorice has already been shown in OVX rat models to reduce bone resorption and osteoclast differentiation, suggesting that licorice flavonoids as a group may be applicable to primary osteoporosis[49]. Overall, current evidence supports LTG as a promising licorice‐derived candidate with combined osteoanabolic and metabolic actions, but its efficacy, optimal dosing and safety in different forms of metabolic and primary osteoporosis still need to be defined in rigorous mammalian and clinical studies.

#### *Morus alba* L.

In addition to Mul-A versus MRC-standardized extracts with CGA as a mass marker, morusinol provides more targeted mechanistic evidence for the role of *Morus alba* L. in OVX-related bone loss. In this study, morusinol was selected from a library of natural compounds by high-throughput virtual screening targeting MSX2, and its anti-osteoclast activity was validated in RANKL-induced BMMs: morusinol reduced the formation of TRAP positive multinucleated osteoclasts and the area of bone resorption lacunar pits, and down-regulated the protein levels of PU.1 and CTSK. More importantly, its effects were directed to the first stage of osteoclast fusion, as shown by the reduction of the proportion of fusion cells and the formation of F-actin ring/closed band, and the inhibition of fusion-related genes. At the mechanistic level, molecular docking suggested a potential interaction between morusinol and key residues of MSX2. Co-IP results showed that the formation of Msx2-PU.1 complex was reduced and the ubiquitination of PU.1 was enhanced. In genetic validation, deletion of Fbxw7 significantly attenuated/reversed the osteoclast inhibitory effect of morusinol, suggesting that it may promote PU.1 degradation through the Msx2-FBxw7-PU.1 axis, thereby inhibiting fusion and limiting osteogenesis. In vivo experiments further showed that morusinol reduced osteoclases-related readout and improved bone microstructure in OVX mice; At the same time, the authors report limited magnitude of changes in inflammation-related transcripts, and a multitarget role cannot be completely ruled out. Based on the available genetic and biochemical evidence, Msx2-Fbxw7-Pu.1 axis may be one of the important mechanisms underlying this effect, but its relative contribution to the overall action spectrum still needs to be further defined [78].

#### *Pueraria lobata* (Willd.) Ohwi

*Pueraria lobata* (Willd.) Ohwi, the dried root of the leguminous plant *Pueraria montana* var. *lobata*, is a classic MFH resource in China [79], where it has long been used both as a traditional remedy and as a food ingredient [139]. Puerarin, a daidzein-8-C glucoside, is the major isoflavonoid in it, and is unique in that it contains C–C conjugated glucose at position 8 of the isoflavonoid structure. Because of its structural stability, it is often used to evaluate its atypical estrogen-like osteoprotective effect in menopause related bone loss models [81].

Across OVX and other postmenopausal osteoporosis–relevant models, the evidence converges on a consistent bone-protective profile of puerarin. Puerarin increases lumbar/femoral bone mineral density and bone mineral content, improves trabecular microarchitecture, and enhances biomechanical performance [26, 121]; it also increases tibial dry and ash weights and mineral content, elevates calcium and phosphorus levels, and reduces bone turnover indices such as serum ALP, suggesting benefit via improved mineral substrate availability and rebalancing of bone remodelling. Relative to oestrogen intervention, its weaker impact on the uterine index implies a degree of tissue selectivity [36]. In fracture-healing models, puerarin improves radiographic healing scores and osteogenic readouts while reducing RANKL expression and the RANKL/OPG ratio, consistent with attenuation of osteoclast predominance [181]. When co-administered with alendronate sodium, further reductions in oxidative stress and inflammatory markers accompany greater BMD gains, supporting a mechanism that enhances standard anti-resorptive therapy primarily by alleviating systemic pathological burden rather than simple on-target additivity with bisphosphonates. Mechanistically, its pro-osteogenic effects align with the miR-21-5p–TGF-β1/Smads axis and pathway inhibition partially blunts skeletal benefits [135]; by contrast, findings involving BMP-2, VEGF, and bone–central protein changes remain largely correlative and require more stringent causal validation [135]. Moreover, there is no clear evidence of estrogen receptor binding and estrogen-dependent cell proliferation, suggesting that its action is independent of the classical estrogen receptor pathway [135]. Other studies have found that it down-regulates TNF-α and C-reactive protein (CRP) without increasing estradiol, which suggests that puerarin mainly exerts anti-inflammatory effects rather than acting as an estrogen replacement [30].

In models of diabetic osteoporosis (DOP), hyperglycaemia and oxidative stress markedly suppress osteoblast function and enhance osteoclast activity. In streptozotocin (STZ)-induced DOP rats, oral puerarin at 80 mg/kg per day significantly increases proximal femoral BMD, restores the orderly arrangement of osteoblasts along trabecular surfaces and reduces the number of osteoclastic resorption lacunae, indicating recovery of impaired osteogenesis together with inhibition of osteoclast formation in a high-glucose milieu [161]. In KKAy mice with type 2 diabetes complicated by obesity and severe islet dysfunction, puerarin not only improves BMD but also lowers MDA, 8-hydroxy-2′-deoxyguanosine (8-OHdG), transforming growth factor-β (TGF-β) and OPN while increasing the activities of SOD, CAT and glutathione peroxidase (GSH-Px). At the same time, it raises fasting insulin and reduces fasting blood glucose and glycated haemoglobin, indicating better glycaemic control and partial restoration of islet function. Together, these changes suggest that alleviation of oxidative stress, improvement of glucose metabolism and repair of the islet microenvironment contribute to a more favourable bone remodelling state [25, 65]. These findings are consistent with results from STZ-induced osteoporotic rats in which puerarin preserves bone mass by suppressing histone deacetylase 1 and 3 (HDAC1/HDAC3) mediated inflammation and apoptosis [25]. In PMOP models, puerarin likewise stabilises bone turnover. In OVX rats displaying sparse, disorganised trabeculae and widened marrow cavities, puerarin increases BMD, reduces serum tartrate-resistant acid phosphatase-5b (TRACP-5b), TNF-α and IL-6, and enhances SOD and GSH-Px activities. Histology shows increased trabecular number and thickness with more regular architecture, indicating that puerarin restores the dynamic balance between osteoblast and osteoclast activity mainly by attenuating inflammation and oxidative stress [94, 171, 176]. Another OVX study demonstrates that puerarin downregulates peroxisome proliferator-activated receptor gamma (PPARγ) and Axin2 while upregulating β-catenin, implicating inhibition of the PPARγ/Axin2 axis and activation of Wnt/β-catenin signalling as mechanisms that promote osteogenic differentiation and suppress bone marrow adipogenesis [8]. Co-administration with zinc further limits OVX-induced marrow fat accumulation and bone loss, hinting that puerarin may potentiate the skeletal benefits of other nutritional factors in regulating the competition between adipogenesis and osteogenesis [68].

Multi-omics and microbiota studies extend these effects along the metabolism-bone interactions. Serum metabolomics in OVX rats shows that puerarin reshapes phospholipid metabolism and polyunsaturated fatty acid biosynthesis, correcting lipid dysregulation while concurrently alleviating bone loss and trabecular damage, in association with Wnt activation and PPARγ inhibition [57]. In OVX mouse models, puerarin remodels gut microbiota composition, increases the production of short-chain fatty acids (SCFAs) and repairs intestinal barrier integrity, thereby improving gut microbiota and creating a more favourable bone microenvironment [57]. In high-fat diet plus STZ-induced type 2 diabetic C57BL/6J mice, pioglitazone aggravates bone marrow adiposity and accelerates bone loss; combination treatment with puerarin promotes alpha-linolenic acid and glycerophospholipid metabolic pathways and rebalances key genera such as *Alloprevotella*, *Fusobacterium* and *Rodentibacter*. These shifts are accompanied by improved trabecular microarchitecture and better glycaemic control, partially offsetting the deleterious skeletal effects of pioglitazone [57].

At the cellular level, puerarin protects osteoblasts and enhances osteogenic differentiation through several signalling routes. In dexamethasone-treated human osteoblasts (hFOB 1.19), puerarin suppresses JNK signalling and activates phosphoinositide 3-kinase (PI3K)/Akt, decreases Bax and cleaved caspase-3, and limits mitochondrial cytochrome c release, thereby mitigating glucocorticoid-induced osteoblast apoptosis and supporting its potential in secondary osteoporosis [162]. In human bone marrow mesenchymal stem cells, puerarin upregulates osteogenic transcription factors Runx2, Osterix and OCN and promotes osteogenic differentiation via nitric oxide/cyclic guanosine monophosphate (NO/cGMP) signalling [77]. Local application of puerarin in a rat calvarial critical-size defect model enhances angiogenesis and new bone formation and accelerates defect repair, suggesting additional utility in bone regeneration and fracture healing[9].

Overall, convergent evidence from animal models, cell systems, metabolomics and microbiota analyses positions puerarin as one of the best characterised MFH-derived modulators of the metabolic-bone interactions. From an osteoporosis perspective, however, large randomised controlled trials with fracture or BMD endpoints in patients with metabolic osteoporosis are still lacking, and the diversity of dosing regimens and disease models, together with limited pharmacokinetic and target-engagement data in humans, means that its optimal therapeutic window, combination strategies with standard antidiabetic or antiosteoporotic agents and long-term skeletal outcomes remain to be clarified.

#### *Sophora japonica* L.

*Sophora japonica* L. is a MFH flower bud traditionally used as a haemostatic agent [40, 116]. Its main chemical constituents include terpenoids, phospholipids, alkaloids, amino acids and fatty acids [23, 32]. Sophoricoside, the characteristic isoflavone glycoside of *S. japonica*, has been shown in in vitro and in vivo studies to markedly reduce osteoclast numbers, inhibit formation of the F-actin sealing zone, and downregulate c-Fos, NFATc1 and the effector genes *Ctsk*, *Mmp9* and *Dcstamp*. In OVX rats, sophoricoside increases BMD and improves trabecular microarchitectural parameters, and it promotes MCF-7 cell proliferation, indicating an oestrogen-like activity [1]. In addition, sophoricoside decreases the expression of inflammatory mediators in lipoteichoic acid (LTA)-stimulated RAW 264.7 cells, primary bone marrow derived macrophages (BMDMs) and primary lung macrophages [140], suggesting that its anti-inflammatory action may represent a shared mechanism underlying its regulation of bone metabolism and anti-osteoporotic effects. Current evidence therefore suggests that sophoricoside acts mainly by inhibiting NFATc1- and c-Fos-driven osteoclast differentiation, while its oestrogen-like and anti-inflammatory properties further favour the restoration of balanced bone remodelling. In addition to sophoricoside, genistein isolated from the flower buds of *Sophora japonica* L. also exhibits clear anti-osteoporotic activity. Studies have shown that large-scale purified genistein derived from *Sophora japonica* L. significantly increases femoral and tibial BMD in OVX rats (achieving a 35–43% recovery at 4 weeks), elevates bone calcium, phosphorus, and magnesium contents, and improves trabecular thickness, area fraction, and number, with an efficacy comparable to that of soy-derived genistein. As a phytoestrogen, genistein exerts protective effects by promoting osteogenesis and reducing bone loss [130].

However, the direct roles of *Sophora japonica* L.–derived isoflavones in glucose and lipid metabolism and mitochondrial function, as well as its contribution to systemic metabolic and skeletal interactions, remain to be defined; further studies in metabolic osteoporosis models and clinical cohorts are needed to clarify its translational relevance. The co-reported metabolic readouts and osteoprotective outcomes of MFH-derived flavonoids are summarized in Table 1.

**Table 1:** MFH-derived flavonoids: co-reported metabolic readouts and osteoprotective outcomes in osteoporosis models

Major category	MFH materials	Active ingredients	Chemical types	OP type	Functions	Mechanisms	References
Flavonoids	*Glycyrrhiza glabra*	Liquiritigenin	Flavonoids	Primary osteoporosis	Reduce osteoclast activity; Prevent bone loss	Auto lysosomal degradation and autophagic flux↑ (Beclin 1↑, LAMP1↑, p62↓); Mitochondria associated apoptosis↓ (Bcl 2↑, Bax↓, cleaved caspase 3↓); Osteoclast number↓ (N.Oc/B.Pm↓)	[9]
	*Glycyrrhiza uralensis* Fisch	Liquiritigenin	Flavonoids	Secondary osteoporosis	Increase BMD; Improve bone strength; Anti-inflammatory	Wnt/β-catenin↑; LRP5↑, p-GSK3β (Ser9)↑; BMD↑	[124]
	*Morus alba* L.	Morusinol	Flavonoids	Primary osteoporosis	Inhibit osteoclast fusion; Promote bone formation; Promote angiogenesis	MSX2–PU.1 axis; FBXW7-mediated PU.1 ubiquitination/degradation↓	[78]
	*Sophora japonica* L.	Sophoricoside	Isoflavone glycosides	Primary osteoporosis	Estrogen-like activity; Improve bone strength; Increase bone formation markers; Inhibit bone resorption	ER signaling; ALP↑, OC↑; ACP↓	[1]
	*Sophora japonica* L.	Genistein	Isoflavones	Primary osteoporosis	Estrogen-like activity; Anti-osteoporosis	Femur/tibia BMD↑; bone Ca/P/Mg↑; trabecular area/thickness/number↑	[130]
	*Astragalus membranaceus* (Fisch.) Bge	Calycosin	Isoflavones	Primary osteoporosis	Promote BMSC autophagy; Promote osteogenic differentiation	PI3K/Akt/mTOR↑; autophagy↑; Runx2↑, ALP↑	[142]
	*Glycine max* (L.) Merr	Daidzein	Isoflavones	Primary osteoporosis	Inhibit osteoclastogenesis; Prevent bone loss; Anti-inflammatory; Anti-oxidation	TNF-α↓; CD4 + CD28null T cells↓; RANKL/OPG balance modulation; ROS↓	[122]
		Daidzin	Isoflavones	Primary osteoporosis	Prevent bone loss; Improve lipid metabolism; Prevent uterine atrophy	Bone turnover↓; serum TC↓, TG↓	[125]
		Genistin	Isoflavones	Primary osteoporosis	Prevent bone loss; Improve lipid metabolism; Prevent uterine atrophy; Prevent bone loss; Inhibit B-lymphopoiesis	Bone turnover↓; serum TC↓, TG↓; BMP-4↑, MAPK10↑; MMP-13↓, IL-1β↓, IL-6↓	
							[125]
				Secondary osteoporosis	Prevent bone loss; Inhibit B-lymphopoiesis;	Bone resorption↓; B-lymphopoiesis regulation	
		Glycitin	Isoflavones	Primary osteoporosis	Prevent bone loss; Improve lipid metabolism; Prevent uterine atrophy	Bone turnover↓; serum TC↓, TG↓	[125]
	*Pueraria lobata* (Willd.) Ohwi	Puerarin	Isoflavones	Primary osteoporosis	Promote osteoblast proliferation and activity; Balance bone metabolism; Inhibit bone resorption; Improve bone microarchitecture; Anti-oxidation; Increase BMD; Improve bone histopathology; Anti-inflammatory; Prevent bone loss; Increase bone mass; Improve bone strength; Promote fracture healing	RANKL/RANK↓, OPG↑; TRAP activity↓, urinary DPD↓, ROS↓; miR-21-5p/TGF-β1/Smads signaling modulation; TNF-α↓, CRP↓; Bone metabolism markers modulation; PPAR-γ↓, Axin2↓, Wnt/β-catenin↑; BMC↑, bone Ca content↑, serum ALP↓; BMD↑, serum ALP↓, urinary Ca/Cr↓, Dpd/Cr↓; BMP-2↑, VEGF↓, BV/TV↑, Tb.N↑, Tb.Th↑; BMD/BMC↑; maximal load↑; stiffness↑ BMD↑, ALP/BGP/PICP/TRAP-5b modulation, TNF-α↓, TGF-β↓, IL-6↓; BMD↑, TRACP5b/RANKL/PINP/BCP modulation, SOD↑, GSH-Px↑, MDA↓, H₂O₂↓; RANK/RANKL/OPG signaling modulation, ALP↑, TRAP↓	[8, 26, 30, 36, 46, 64, 81, 94, 121, 135, 171, 176]
				Secondary osteoporosis	Prevent bone loss; Improve glycemic control; Modulate gut microbiota; Improve lipid metabolism Increase BMD; Anti-oxidation; Improve glucose metabolism; Regulate calcium metabolism; Improve cardiovascular profile; Increase BMD, Hypoglycemic effect; Anti-apoptosis (osteoblasts)	ALP↑, mineralized nodules↑, Runx2↑, BMP-2↑, TRAP↓, gut microbiota and α-linolenic acid/glycerophospholipid metabolism regulation, TNF-α↓, IL-1β↓; BMP-6 and VEGF modulation; HDL-C↑, LDL-C↓, TG↓; Osteoblasts↑, osteoclasts↓; p-JNK↓, p-Akt↑, Bax/Bcl-2 ratio↓, caspase-3 activation↓;	[63, 65, 155, 161, 162]

### Polysaccharides

#### *Astragalus membranaceus* (Fisch.) Bge.

In addition to isoflavones, Astragalus polysaccharide (APS) extracted from *Astragalus membranaceus* (Fisch.) Bge. is one of the most extensively studied constituents with immunomodulatory and haematopoietic activities, and has recently attracted attention for its actions on bone metabolism in a metabolic stress context. In OVX rats, oral administration of APS at 400 mg/kg per day for 12 weeks significantly improves bone mass and trabecular microarchitecture, indicating that it can reverse cancellous bone loss and trabecular rarefaction induced by oestrogen deficiency. Serum analysis shows that ALP and OCN levels are elevated in the OVX group, reflecting a high bone turnover state, whereas APS treatment lowers ALP and OCN while increasing serum calcium, suggesting that excessive bone remodelling is restrained and calcium incorporation into bone is enhanced, thereby helping to restore bone mineral metabolic balance. Protein expression studies further demonstrate that the BMP-2/Smads pathway is inhibited in OVX bone, and that APS upregulates BMP-2, phosphorylated Smad1 and phosphorylated Smad5, thereby promoting osteogenic differentiation and increasing bone formation efficiency, which provides a molecular basis for correcting the imbalance in bone remodelling [144]. Other work has shown that APS effectively alleviates iron overload-induced dysfunction of BMSCs by limiting ROS accumulation, preserving proliferative capacity, inhibiting apoptosis and senescence and maintaining the expression of pluripotency-associated genes [154].

These findings suggest that APS helps BMSCs to cope with oxidative and iron-related metabolic stress and to maintain a more favourable local microenvironment for osteogenesis. Taken together, APS appears to regulate bone metabolism under conditions of oxidative and iron overload stress and to exert antioxidant and anti-osteoporotic effects through several complementary mechanisms, although further well-designed experimental and translational studies are required to define its key molecular targets, dose–response characteristics and relevance to metabolically driven osteoporosis in humans.

#### *Glycine max* (L.) Merr.

*Glycine max* (L.) Merr. is not only rich in isoflavones but also provides water-soluble soybean fiber (WSSF), a polysaccharide fraction of medicine-food homology origin with dual metabolic effects on lipid regulation and mineral absorption. In a bilateral OVX model of female Sprague–Dawley (SD) rats, feeding an experimental diet containing 5% WSSF for 4 weeks simultaneously improved calcium absorption, bone mineral content and the blood lipid profile. Compared with sham-operated controls, OVX rats showed a marked reduction in apparent calcium absorption and femoral calcium content, whereas supplementation with WSSF significantly restored both indices, suggesting that it attenuates oestrogen deficiency-related bone loss by promoting intestinal calcium absorption and increasing calcium deposition in bone. With respect to lipids, serum TC levels were significantly higher in the OVX group than in sham-operated rats, while WSSF markedly blunted the OVX-induced rise in TC and partially corrected the unfavourable changes in LDL/VLDL and HDL, thereby improving dyslipidaemia under oestrogen-deficient conditions. Analysis of caecal contents further showed that WSSF increased caecal content mass, lowered caecal pH and significantly elevated the levels of acetic acid, propionic acid and other SCFAs, while increasing the proportion of soluble calcium and enhancing colonic calcium absorption[84]. These findings indicate that water-soluble soybean fiber acts primarily as a fermentable dietary fibre that is metabolised by the colonic microbiota to SCFAs, which in turn acidify the local environment, increase calcium solubility and improve systemic calcium utilisation, with concurrent benefits on cholesterol metabolism. Overall, WSSF appears to exert osteoprotective effects by enhancing intestinal calcium absorption and improving calcium utilisation, together with partial correction of dyslipidaemia, illustrating how MFH-derived polysaccharides can coordinately modulate calcium handling and lipid metabolism.

#### *Lycium barbarum* L.

*Lycium barbarum* L. is a member of the Solanaceae family and is regarded in TCM as strengthening tendons and bones, it is also frequently prescribed to improve male reproductive function [129]. Modern pharmacological studies have shown hepatoprotective, neuroprotective and anti-ageing activities [29, 148]. Among its constituents, *Lycium barbarum* polysaccharides (LBP) and *Lycium barbarum* polysaccharide-glycoprotein complexes (LBP-glycoprotein, LBP-GP) are the best characterised [21]. Current experimental data indicate that these fractions attenuate lipotoxic and stress-related damage in osteoblasts and improve bone remodelling in inflammatory bone loss models. In fatty acid-induced osteoblastic injury, LBP shows a pronounced cytoprotective effect. In MC3T3-E1 osteoblast-like cells, palmitate (PA) reduces cell viability and increases apoptosis, accompanied by induction of endoplasmic reticulum stress (ERS) markers glucose-regulated protein 78 (GRP78) and C/EBP homologous protein (CHOP), upregulation of caspase-3, caspase-9 and caspase-12, and enhanced phosphorylation of JNK. Co-treatment with LBP restores cell viability in a concentration-dependent manner, lowers the apoptotic rate and downregulates ERS-related proteins and p-JNK. These findings suggest that LBP protects osteoblasts in a lipotoxic milieu mainly by dampening activation of the ERS/JNK axis and thereby limiting mitochondria-dependent apoptosis [48]. Perimenopausal and postmenopausal osteoporosis models highlight an additional connection between LBP and intestinal metabolism. In an in vitro colonic fermentation system inoculated with faecal samples from patients with postmenopausal osteoporosis, the addition of LBP increases total SCFAs, with higher levels of acetate, propionate and butyrate. Fermentation supernatants applied to MC3T3-E1 cells under osteogenic induction promote cell proliferation, enhance ALP activity and OCN secretion, and upregulate osteogenic genes. These results support a functional axis whereby LBP-driven modulation of the gut microbiota and SCFA production is translated into improved osteoblast activity [60].

Beyond lipotoxic injury, LBP has also been shown to counteract toxicant associated osteogenic impairment in a cadmium exposed BMSC model. Cadmium disrupted autophagic flux by limiting autophagosome formation and reducing autolysosome generation, with features consistent with impaired LC3 conversion and loss of p62 degradation, whereas LBP reactivated the autophagy process and facilitated autophagosome and autolysosome formation. Functionally, this restoration of autophagy was accompanied by improved cell viability and attenuation of cadmium induced suppression of osteogenic differentiation, supporting autophagy modulation as a key mechanism by which LBP preserves osteogenic capacity under secondary stress conditions relevant to environmentally driven bone loss [60]. Other studies have found that EBP pretreatment has a positive protective effect on osteoblast formation during the progression of glucocorticoid-induced ossification, and its mechanism may be to reduce osteoblast apoptosis by up-regulating PI3K/AKT/mTOR and Wnt/β-catenin signaling pathways and inhibiting mitochondrial apoptosis of osteoblasts [173].

Studies on LBP-GP focus mainly on inflammation-related bone loss. LBP-GP treatment increases ALP activity and mineralised nodule formation and upregulates osteogenic marker genes, while phosphorylation of ERK1/2 is enhanced. The use of an ERK inhibitor partially abolishes these effects, indicating that LBP-GP promotes osteogenic differentiation through the ERK1/2 pathway. In parallel, LBP-GP reduces the levels of TNF-α and IL-1β and decreases inflammatory cell infiltration, consistent with concomitant relief of inflammation and suppression of bone resorption [55]. Overall, LBP and LBP-GP position *Lycium barbarum* L as a promising MFH candidate for complex metabolic-bone phenotypes, with actions that extend from protection against lipotoxic stress in osteoblasts to modulation of gut-derived metabolites and inflammatory bone loss. Evidence, however, is still largely confined to cellular work and a limited range of preclinical models, and well-designed studies in metabolically driven osteoporosis, followed by early clinical trials, will be needed to define their effective exposure ranges, safety and translational relevance.

#### *Nelumbo nucifera* Gaertn.

*Nelumbo nucifera* Gaertn. is a classic MFH material that has long been used in diets and TCM to dispel summer heat, resolve dampness and regulate body weight and lipids [103]. Its polysaccharide fraction has recently been shown to have potential in the management of osteoporosis. In female C57BL/6 mice, bilateral OVX combined with a high-fat diet (HFD) was used to exacerbate both bone loss and lipid metabolic disturbance. On this background, oral administration of a pectinase-derived lotus leaf extract polysaccharide, LLEP (30 or 100 mg/kg per day) for 4 weeks markedly attenuated cancellous bone damage associated with oestrogen deficiency plus HFD. Micro-CT revealed that mice receiving the higher dose of LLEP exhibited significantly improved trabecular microarchitectural indices compared with OVX + HFD controls, indicating a partial reversal of oestrogen deficiency-induced deterioration of bone structure. With respect to bone turnover markers, LLEP significantly reduced serum levels of C-terminal telopeptide of type I collagen (CTX-1), whereas its effect on N-terminal propeptide of type I procollagen (PINP) was relatively inconsistent. The authors proposed that at five weeks after OVX this observation window may be too short for biochemical markers of bone formation to fully capture the ongoing reconstruction of trabecular bone, and longer-term studies are needed to clarify the sustained impact of LLEP on the formation phase. In terms of body weight and fat deposition, LLEP partly suppressed the progressive weight gain and peri-gonadal fat accumulation induced by OVX plus HFD, suggesting an auxiliary role in alleviating energy surplus and adipose tissue expansion. At the cellular level, LLEP exerts a direct anti-resorptive action on osteoclastogenesis. It significantly inhibits the differentiation of osteoclasts derived from BMMs cultured with macrophage colony-stimulating factor (M-CSF) and receptor activator of RANKL, reducing osteoclast formation without markedly affecting cell viability. Mechanistically, LLEP downregulates the RANKL-induced master transcription factors c-Fos and NFATc1, and suppresses the transcription of downstream osteoclast-associated genes, thereby blocking osteoclast formation and bone resorption [37].

Taken together, lotus leaf polysaccharides appear to protect bone in OVX + HFD mice by inhibiting osteoclastogenesis while partly attenuating diet-induced weight gain and fat accumulation, a pattern that is relevant to oestrogen-deficient states with coexisting dyslipidaemia and osteoporosis. However, current evidence is limited to a single short-term OVX + HFD mouse model, and the mechanisms by which LLEP modulates systemic lipid metabolism remain largely undefined.

#### *Polygonatum sibiricum* Red. and *Polygonatum cyrtonema* Hua

*Polygonatum* spp. are widely used MFH rhizomes in traditional Chinese medicine, with more than two millennia of recorded use. They are traditionally prescribed to tonify Qi and Yin, nourish the kidney and spleen and enhance vitality, and are used for fatigue, age-related frailty, memory decline and metabolic disturbances [11, 67, 69, 166, 168, 170]. The classical processing method of “nine cycles of steaming and drying” is believed to reduce toxicity and enhance efficacy, and processed products are therefore commonly used in clinical practice [167]. Polysaccharides are considered the principal functional constituents of *Polygonatum*, with *Polygonatum sibiricum* polysaccharide (PSP) and *Polygonatum cyrtonema* polysaccharide (PCP) identified as key material bases for its anti-osteoporotic and metabolic regulatory effects. Available animal and cell studies have mainly focused on PMOP, osteoporotic fracture, and diabetes-related bone loss, and collectively characterise *Polygonatum* polysaccharides in terms of coordinated effects on bone remodelling and glucose and lipid metabolism.

In OVX rats, oral administration of PSP at 100–400 mg/kg for 35 days increases whole-body and long-bone BMD, and improves trabecular microarchitecture, with denser and more regularly arranged trabeculae than in untreated OVX controls. Serum BGP, bone-specific alkaline phosphatase (BALP) and TRAP levels are reduced, whereas BMP and basic fibroblast growth factor (bFGF) expression in bone tissue is upregulated. These changes indicate that PSP, in the absence of exogenous oestrogen supplementation, corrects a high bone turnover state while activating osteogenesis-related growth factors, thereby improving the quality of bone formation and remodelling [167]. In a separate study, PSP at 500–1000 mg/kg and an oestrogen comparator both increased proximal femoral BMD and the maximal elastic load in tibial three-point bending tests, decreased TRAP and PINP, elevated OPG and BGP and enhanced G protein-coupled receptor 48 (GPR48) and BMP-2 expression in tibia. These findings suggest that PSP improves fracture healing quality by modulating bone metabolic factors and the GPR48/BMP-2 axis, and to some extent confers bone benefits comparable to oestrogen replacement [112]. The anti-osteoporotic efficacy of PSP has also been demonstrated in rat models of DOP [152].

At the cellular and local bone loss levels, PSP exerts both pro-osteogenic and anti-resorptive actions. In mouse BMSCs, PSP at 10–50 mg/L increases ALP activity and mineralised nodule formation without impairing cell viability, upregulates osteogenic marker genes such as *Col1*, *Runx2* and *Ocn* and promotes nuclear accumulation of β-catenin, while having only limited effects on BMP signalling. In a receptor activator of RANKL-induced osteoclast differentiation model, PSP inhibits the formation of TRAP^+^ multinucleated osteoclasts in a dose-dependent manner and downregulates osteoclast-associated genes including *Trap*, *Mmp9*, *cathepsin K (CTSK)* and *Nfatc1*. In a lipopolysaccharide (LPS)-induced calvarial osteolysis model, PSP reduces trabecular destruction and osteoclast accumulation. Together, these results indicate that PSP promotes osteogenic differentiation primarily through activation of the Wnt/β-catenin pathway, while concomitantly attenuating RANKL/NFATc1-driven osteoclastogenesis and inflammatory bone resorption [18].

In metabolically driven osteoporosis models, *Polygonatum* polysaccharides also show multi-target regulation of glucose-lipid metabolism and inflammatory responses. In a STZ-induced type 2 diabetic zebrafish model, PSP lowers blood glucose, enhances tail fin regeneration and restores bone structure. Non-targeted metabolomics reveal that PSP modulates arachidonic acid, linoleic acid and alpha-linolenic acid metabolism and affects PPAR signalling, steroid hormone biosynthesis, selected amino acid pathways and sphingolipid metabolism. In parallel, PSP reduces the transcription of inflammatory cytokines such as TNF-α and IL-1β [13]. These findings suggest that PSP reshapes the bone-related internal milieu under hyperglycaemic conditions through reprogramming of lipid and amino acid metabolism combined with anti-inflammatory actions.

In a zebrafish larval model of “type 2 diabetes with concomitant osteoporosis” established by STZ microinjection followed by prednisolone immersion, PCP at 60–240 μg/mL reduces tissue glucose content, restores skull mineralisation area and BMD and upregulates mRNA expression of osteogenic and matrix-related genes including *Runx2b*, *Sp7*, *Col1a2*, *Sparc* and *Vdrb*. This profile indicates that, in this composite phenotype characterised by hyperglycaemia and bone loss, PCP both lowers glucose burden and strengthens the osteogenic gene programme, thereby exerting a direct and bidirectional effect on metabolically driven bone loss [164].

Overall, *Polygonatum* polysaccharides combine regulation of bone metabolism with broader metabolic reprogramming. They restore the balance between osteogenesis and osteoclastogenesis through pathways such as Wnt/β-catenin and GPR48/BMP-2, while concurrently modulating lipid and amino acid metabolism and mitigating inflammation and hyperglycaemic stress to optimise the bone-related microenvironment. These convergent preclinical data support the potential of *Polygonatum* polysaccharides as MFH-derived candidates for the management of metabolic osteoporosis.

#### *Portulaca oleracea *L*.*

*Portulaca oleracea* L*.* is a MFH material with a long history of use, first documented in *Ben Cao Gang Mu* (Compendium of Materia Medica). Traditionally, it is used to clear heat, detoxify and cool the blood to stop bleeding [106]. Its polysaccharide fraction has been shown to possess marked antioxidant and immunomodulatory activities. A Viscozyme-assisted extract of P. oleracea yields an acidic heteropolysaccharide designated VPOP1, with a molecular weight of approximately 7.6 kDa, which exhibits pronounced “anti-oxidative stress and osteoblast-protective” effects in osteoblastic cells and zebrafish models. H₂O₂-induced oxidative injury of MC3T3-E1 osteoblastic cells, VPOP1 at 25–100 μg/mL significantly improves cell viability, suppresses excessive production of ROS and alleviates mitochondrial pathway-mediated apoptosis by upregulating Bcl-2 and downregulating Bax, caspase-3 and cytochrome c (CytoC). Consistent with these cytoprotective effects, Alizarin Red S staining shows a clear recovery in both the number and area of mineralised nodules. In an in vivo zebrafish embryo model, H₂O₂ is used to induce impaired bone mineralisation and calcein staining to assess bone formation; pre-treatment with VPOP1 markedly reverses H₂O₂-induced loss of bone fluorescence and defective mineral deposition, indicating protection against oxidative stress-related bone loss at the whole-organism level. Non-targeted metabolomics based on UPLC-Q-Orbitrap-HRMS in zebrafish further elucidates the metabolic basis of VPOP1 action. VPOP1 significantly normalises 28 potential metabolic biomarkers disrupted by H₂O₂, among which leukotriene A₄/D₄, L-dopa and L-tyrosine are closely linked to inflammatory lipid mediators and catecholamine metabolism. Pathway enrichment analysis indicates that arachidonic acid metabolism, tyrosine and phenylalanine metabolism and sphingolipid metabolism are the principal metabolic routes modulated by VPOP1, all of which are tightly associated with oxidative stress, inflammatory responses and membrane lipid homeostasis. These findings suggest that *P. oleracea* polysaccharides mitigate apoptosis and functional impairment of osteoblasts under oxidative stress by remodelling oxidative and inflammatory lipid signalling as well as aromatic amino acid metabolism, thereby preserving bone-forming capacity [163].

From a metabolic-bone perspective, VPOP1 does not primarily act through the classical RANKL-dependent osteoclastogenic pathway, but instead exerts its effects via antioxidant, anti-apoptotic and metabolically reprogramming mechanisms, offering an MFH-based polysaccharide strategy for metabolic osteoporosis under conditions of heightened oxidative stress. At present, however, evidence for VPOP1 is confined to short-term zebrafish and osteoblast models, and its pharmacokinetics, actions on osteoclast and immune compartments and efficacy in mammalian osteoporosis models remain essentially unknown. The co-reported metabolic readouts and osteoprotective outcomes of MFH-derived polysaccharides are summarized in Table 2.

**Table 2:** MFH-derived polysaccharides: co-reported metabolic readouts and osteoprotective outcomes in osteoporosis models

Major category	MFH materials	Active ingredients	Chemical types	OP type	Functions	Mechanisms	References
Polysaccharides	*Astragalus membranaceus* (Fisch.) Bge	Astragalus polysaccharide	Polysaccharides	Primary osteoporosis	Preserve BMD and bone mass	BMP-2/Smads signaling↓	[144]
	*Glycine max* (L.) Merr	Water-soluble soybean fiber		Primary osteoporosis	Prevent bone loss; Anti-hypercholesterolemia	Ca absorption↑; femoral Ca content↑; TC↓, HDL↓	[84]
	*Lycium barbarum* L	Lycium barbarum polysaccharide		Primary osteoporosis	Promote osteoblast proliferation and differentiation; Modulate gut microbiota metabolism	ALP activity↑; OCN↑; BMP-2/RUNX2 mRNA↑; SCFAs↑	[60]
				Secondary osteoporosis	Attenuate cadmium related osteogenic suppression; improve BMSC viability	Autophagic flux↑ (LC3 II↑, Beclin 1↑, P62↓), Osteogenesis↑ (ALP↑, BMP2↑, COL1↑, RUNX2↑), Apoptosis↓ (BAX↓, Caspase 3↓, BCL2↑) PI3K/Akt/mTOR phosphorylation↑; Wnt/β-catenin (Lrp5, β-catenin, Runx2, Osx)↑; cleaved caspase-3↓, Bax↓; Bcl-2↑	[60, 173]
				Secondary osteoporosis	Promote osteoblast proliferation and differentiation; Anti-apoptosis		
	*Nelumbo nucifera* Gaertn	Lotus leaf polysaccharide		Primary osteoporosis	Inhibit osteoclastogenesis; Reduce bone loss; Anti-obesity	RANKL-induced c-Fos/NFATc1↓; body weight and fat accumulation↓	[37]
	*Polygonatum cyrtonema* Hua	Polygonatum polysaccharide		Secondary osteoporosis	Hypoglycemic effect; Anti-osteoporosis; Anti-oxidation	Runx2b↑, Sp7↑, vdrb↑, sparc↑, col1a2 mRNA↑; bone mineralized area↑	[164]
	*Polygonatum sibiricum* Red	Polygonatum polysaccharide		Secondary osteoporosis	Balance bone metabolism; Increase femoral BMD; Regulate blood glucose	OPG/RANKL signaling modulation	[152]
				Primary osteoporosis	Improve bone strength; Increase BMD	GPR48↑, BMP-2↑; ALP↑, OPG↑, BCP↑; TRAP↓, PINP↓	[112]
				Primary osteoporosis	Promote fracture healing	OCN↓, BGP↓, TRAP↓	[22]
				Secondary osteoporosis	Hypoglycemic effect; Anti-inflammatory; Modulate metabolic pathways; Improve osteoporosis	Tissue regeneration↑ (caudal fin length↑); TNF-α↓, IL-1β↓; arachidonic acid/PPAR/α-linolenic acid metabolism regulation	[13]
				Primary/secondary	Promote bone formation; Inhibit osteoclastogenesis	Wnt/β-catenin↑ → Runx2↑, ALP↑, OCN↑; NFATc1↓, MMP-9↓, TRAP↓, CtsK↓	[18]
		Polygonatum sibiricum polysaccharide		Primary osteoporosis	Reverse bone loss; Regulate bone metabolism; Anti-inflammatory	BMP↑; bFGF↑; BGP↓; BALP↓; TRAP↓; TNF-α↓	[165]
	*Portulaca oleracea* L	Viscozyme-assisted POP active fraction		Secondary osteoporosis	Anti-osteoporosis; Anti-oxidation; Anti-apoptosis (osteoblasts)	Bcl-2↑; Bax↓; caspase-3↓; CytoC↓; arachidonic acid/tyrosine/phenylalanine/sphingolipid metabolism regulation	[163]

### Phenolic and polyphenolic compounds

#### *Angelica sinensis* (Oliv.) Diels.

*Angelica sinensis* (Oliv.) Diels. is an important medicinal herb in TCM, noted for its broad therapeutic actions such as nourishing the blood, relieving pain and treating menstrual disorders including irregular menstruation and amenorrhoea [66, 133]. The volatile aromatic phenol guaiacol has been identified as a key small molecule contributing to the anti-resorptive activity of *Angelica sinensis* (Oliv.) Diels. In vitro and in vivo studies show that guaiacol directly disrupts the interaction of RANK with TRAF6 and c-Src, thereby inhibiting RANKL-induced activation of NF-κB, MAPK and AKT signalling. It also markedly suppresses RANKL-triggered intracellular Ca^2^⁺ oscillations and the activation and nuclear translocation of NFATc1 [175]. Consistently, a hydroalcoholic extract of *A. sinensis* inhibits osteoclast formation and activation of p38, ERK, c-Jun N-terminal kinase (JNK) and NF-κB in BMMs, and downregulates *c-Fos, c-Jun, Nfatc1* and downstream genes such as *Trap* and *Oscar*, indicating that guaiacol is likely to constitute an important material basis for the anti-resorptive effect of this herb [52]. However, more rigorously designed clinical studies are still needed to determine whether this MFH-derived phenolic can be safely and effectively translated into therapy for metabolic osteoporosis.

#### *Cinnamomum cassia* Presl

*Cinnamomum cassia* Presl is a typical MFH aromatic herb in TCM, considered warm in nature and entering the heart, lung and bladder meridians, where it is used to dispel cold, relieve pain and promote blood circulation. Modern pharmacological studies indicate that the tannin-type polyphenol corilagin is one of the major active constituents contributing to the bone-protective effects of *C. cassia*. In RANKL-stimulated bone marrow-derived osteoclast precursors and RAW 264.7 cells, corilagin inhibits the formation of TRAP^+^ multinucleated osteoclasts and the development of resorption pits in a dose-dependent manner, downregulates key osteoclastogenic genes including *Nfatc1*, *Ctsk* and *Dcstamp*, reduces RANKL-induced ROS production and suppresses phosphorylation of NF-κB p65 and PI3K/Akt [72]. Collectively, these findings support an anti-resorptive profile associated with attenuation of redox and inflammatory signalling. Whether corilagin also improves systemic metabolic abnormalities and whether such effects translate into skeletal benefits in metabolically driven osteoporosis remain to be established in models capturing both metabolic and bone endpoints.

#### *Curcuma longa* L.

*Curcuma longa* L. is a representative MFH material that is widely used as a culinary spice and as an adjuvant remedy in traditional Chinese medicine for disorders of the respiratory, digestive, circulatory and cutaneous systems [118]. Its rhizomes contain a variety of active components such as polyphenols and terpenoids, among which curcumin, the most representative one, has been shown to have regulatory potential on bone metabolism and related metabolic abnormalities in preclinical studies.

With regard to skeletal effects, curcumin has been investigated predominantly in ovariectomy (OVX)-induced models of postmenopausal osteoporosis (PMOP). In these models, 12-week administration increases bone mineral density (BMD), enhances trabecular number and thickness and improves overall trabecular architecture. These structural benefits are accompanied by up-regulation of osteoprotegerin (OPG) and down-regulation of receptor activator of nuclear factor kappa-B ligand (RANKL), leading to a higher OPG/RANKL ratio, reduced osteoclast activity and restoration of the balance between bone formation and resorption [96]. In senile osteoporosis models, approximately 12 weeks of treatment further increases bone volume/tissue volume (BV/TV), reduces trabecular separation and decreases both bone-marrow adipocytes and tartrate-resistant acid phosphatase (TRAP)-positive osteoclasts [136]. Together, these data suggest that curcumin promotes osteogenesis while suppressing marrow adipogenesis and osteoclastogenesis, thereby improving bone mass and microarchitectural quality through modulation of the bone-marrow microenvironment.

At the level of metabolic and skeletal interactions, curcumin reinforces its osteoprotective actions mainly by attenuating oxidative stress and chronic inflammation. Activation of the forkhead box O3a (FoxO3a)/Wnt signalling pathway has been shown to stimulate osteogenic differentiation, while reductions in ROS, increases in the activities of SOD and CAT, and decreases in the lipid peroxidation marker MDA contribute to the re-establishment of redox homeostasis in bone tissue [97]. In fracture-healing models under osteoporotic conditions, curcumin inhibits the stimulator of interferon genes (STING)/NF-κB inflammatory axis, lowers concentrations of interleukin-6 (IL-6), tumour necrosis factor-α (TNF-α) and osteoclast-related markers, accelerates the transition from cartilaginous to bony callus and improves the mechanical strength of the fracture site [97]. Taken together, curcumin has the potential to improve bone mass and quality, but its efficacy in metabolic abnormalities associated with osteoporosis still needs to be confirmed in rigorously designed clinical trials [157].

#### *Morus alba* L.

Mori Radicis Cortex (MRC), the dried root bark of *Morus alba* L., is a typical MFH herb traditionally credited with clearing lung heat, reducing blood pressure and calming the mind, while mulberry leaves are commonly used for the treatment of wind-heat, common cold and cough [10]. Among its constituents, the stilbenoid glycoside mulberroside A (Mul-A), isolated from the twigs and root bark of *M. alba*, is a structurally defined small molecule. In vitro and in vivo studies, it is mainly manifested as selective inhibition of osteoclast with limited effect on osteogenesis. In RANKL-induced bone marrow-derived osteoclast precursors, Mul-A markedly reduces the number of TRAP^+^ multinucleated osteoclasts and the area of resorption pits. These changes are accompanied by down-regulation of osteoclast-related genes such as nuclear factor of activated T cells 1 (*Nfatc1*), cathepsin K (*Ctsk*) and *Trap*, as well as suppression of microphthalmia-associated transcription factor (Mitf) expression and its nuclear translocation. Mul-A also limits autophagic flux by preventing LC3-II accumulation and p62 degradation, which further attenuates osteoclast differentiation and resorptive function. In OVX mice, Mul-A improves bone volume/tissue volume (BV/TV), trabecular number (Tb.N) and trabecular thickness (Tb.Th) in a dose-dependent manner and reduces the number of osteoclasts along trabecular surfaces, whereas its effects on osteoblast activity and mineral apposition rate are minimal, suggesting that Mul-A primarily mitigates bone loss induced by oestrogen deficiency through Mitf-related autophagy and selective inhibition of osteoclastogenesis [150]. Chlorogenic acid (CGA) is a representative phenolic acid in MRC and serves as a key quality control marker for this crude drug [150]. An MRC extract standardised to CGA improves femoral morphometric parameters in OVX rats, significantly lowers serum ALP and TRAP levels, and reduces the immunohistochemical signal of NFATc1 in femoral tissue. In RANKL-stimulated RAW 264.7 cells, MRC at non-cytotoxic concentrations suppresses TRAP activity and F-actin ring formation, and down-regulates NFATc1, c-Fos and several downstream genes involved in osteoclast differentiation, fusion and matrix degradation, including *Oscar* (osteoclast-associated receptor, OSCAR), *Atp6v0d2* (ATPase H⁺ transporting V0 subunit d2, ATP6V0D2) and *Mmp9* (matrix metalloproteinase 9, MMP-9). These findings indicate that a CGA-rich MRC extract attenuates osteoclast formation and bone resorption mainly by blocking the NFATc1/c-Fos axis and its downstream effector molecules [34]. In addition, CGA has been shown to activate p21 and the nuclear factor erythroid 2-related factor 2/heme oxygenase-1 (Nrf2/HO-1) antioxidant pathway, thereby reducing intracellular ROS, hydrogen peroxide (H₂O₂) and excessive mitochondrial superoxide production, which ultimately inhibits dexamethasone-induced apoptosis and slows the progression of osteoporotic changes [27].

Taken together, Mul-A appears to selectively restrain osteoclast maturation via Mitf-related autophagy, whereas CGA mainly suppresses osteoclastogenesis mediated by NFATc1 and c-Fos while relieving oxidative and metabolic stress. Further studies are needed to clarify their precise molecular targets and translational relevance in metabolic osteoporosis.

#### *Sesamum indicum* L.

Sesame (*Sesamum indicum* L.) is an erect annual herb widely used as a food and edible oil source [7], and in TCM it is prescribed to relieve intestinal dryness and to nourish the liver and kidney. Modern pharmacological studies further document antidiabetic, antioxidant, cardioprotective, antimicrobial and analgesic activities [82]. The key lignan sesamin is regarded as the principal constituent involved in bone regulation. It drives bone marrow mesenchymal stem cells toward the osteogenic lineage, upregulates *Runx2* (runt-related transcription factor 2, Runx2), *Alp*, *Ocn* and *Bmp2* (bone morphogenetic protein 2, BMP2), and activates the Wnt/β-catenin signalling pathway, thereby enhancing osteoblast function. Regarding bone resorption, sesamin suppresses RANKL-induced osteoclastogenesis by inhibiting NF-κB p65 nuclear translocation, downregulating *Ctsk* and *Acp5*, and reducing the formation of TRAP^+^ osteoclasts, which helps to restore bone remodelling balance. The long non-coding RNA DANCR participates in this process and mediates the bidirectional control of Wnt/β-catenin and NF-κB by sesamin; experimental overexpression of DANCR diminishes the magnitude of sesamin-induced bone protection [159]. Taken together, sesamin appears to promote bone formation via Wnt/β-catenin, restrain inflammatory osteoclastogenesis through NF-κB and DANCR, although its efficacy in human metabolic osteoporosis still needs to be verified in well-designed studies. The co-reported metabolic readouts and osteoprotective outcomes of MFH-derived phenolic and polyphenolic compounds are summarized in Table 3.

**Table 3:** MFH-derived phenolics and polyphenols: co-reported metabolic readouts and osteoprotective outcomes in osteoporosis models

Major category	MFH materials	Active ingredients	Chemical types	OP type	Functions	Mechanisms	References
Phenolic and polyphenolic compounds	*Curcuma longa* L.	Curcumin	Polyphenols	Primary osteoporosis	Promote osteogenesis; Inhibit bone resorption; Anti-inflammatory; Anti-oxidation	FoxO3a/Wnt pathway: FoxO3a↓, β-catenin↑, MDA↓, ROS↓; SOD↑, CAT↑; Bone marrow adipocytes↓; STING/NF-κB pathway: STING↓, NF-κB↓; IL-6↓, TNF-α↓; OPG/RANKL signaling modulation	[72]
	*Morus alba* L.	Mulberroside A	Stilbenoid glycosides	Primary osteoporosis	Inhibit osteoclastogenesis; Regulate autophagy	Mitf expression/nuclear translocation↓; autophagy regulation	[150]
		Chlorogenic acid	Phenolic acids		Inhibit osteoclastogenesis; Regulate autophagy	NFATc1/c-Fos signaling↓	[150]
	*Cinnamomum cassia* Presl	Corilagin	Tannins	Primary osteoporosis	Inhibit osteoclastogenesis; Anti-inflammatory; Anti-oxidation	NF-κB↓, PI3K/Akt↓; ROS↓	[72]
	*Sesamum indicum* L.	Sesamin	Lignans	Primary osteoporosis	Promote osteogenic differentiation; Inhibit osteoclastogenesis; Anti-inflammatory	Wnt/β-catenin↑; NF-κB↓	[159]
	*Angelica sinensis* (Oliv.) Diels	Guaiacol	Phenols	Primary osteoporosis	Inhibit osteoclastogenesis; Inhibit bone resorption	RANKL–TRAF6/C-Src interaction↓; NF-κB↓, MAPK↓, AKT↓	[175]

### Terpenoids

#### *Angelica sinensis* (Oliv.) Diels.

*Angelica sinensis* (Oliv.) Diels. is a traditional medicinal and edible herb whose roots are widely used to nourish and invigorate the blood and to treat gynaecological disorders. Its pharmacological activities include haematopoietic, anti-inflammatory, immunomodulatory, antifibrotic and antitumour effects [17, 102, 166, 168, 170]. Among its constituents, the phthalide ligustilide (LIG) has been identified as an important active compound in the context of bone protection. In OVX rats, LIG increases femoral BMD and reduces serum CTX-1 and Dpd without affecting uterine index or serum E₂[62], suggesting that it improves bone health by correcting high bone turnover rather than acting as a classical oestrogen substitute. In a prednisolone-induced osteoporotic zebrafish model and in MC3T3-E1 osteoblast-like cells and BMSCs exposed to H₂O₂, LIG restores vertebral mineralisation, increases ALP activity and mineralised nodule formation and reduces oxidative stress-induced osteoblast apoptosis. Mechanistically, these effects are associated with upregulation of Bcl-2 and activation of G protein-coupled oestrogen receptor 30 (GPR30)/epidermal growth factor receptor (EGFR)/ERK and PI3K/Akt signalling pathways [153]. Taken together, LIG effectively intervenes along a pathological axis in which glucocorticoid exposure and oxidative stress initiate osteoblast injury that culminates in osteoporosis, thereby exerting a clear bone-protective effect, although further studies are still needed to consolidate these findings and define its therapeutic window.

#### *Cornus officinalis* Sieb. et Zucc.

*Cornus officinalis* Sieb. et Zucc. (family Cornaceae) has been used in traditional Chinese medicine for nearly two millennia, with its earliest record in the classic *Shen Nong Ben Cao Jing*. In traditional theory it is sour, astringent and warm in nature, and is used to tonify the liver and kidney and to secure essence and reduce urination; clinically it is prescribed for dizziness and tinnitus, weakness and soreness of the lumbar region and knees, seminal emission, spontaneous sweating and related symptoms [14, 15]. The iridoid glycosides of *C. officinalis* are regarded as the principal pharmacologically active fraction, and modern studies have demonstrated prominent anti-inflammatory, antioxidant, anti-apoptotic and immunomodulatory properties [14, 15]. In OVX rats, 60 days of oral administration of total glycosides of *C. officinalis* significantly increases serum ALP and BGP, while 24-h urinary deoxypyridinoline (Dpd) is markedly reduced. These changes indicate that, under oestrogen-deficient conditions, total glycosides of *C. officinalis* enhance bone formation and suppress bone resorption, shifting bone remodelling from a high-turnover state towards a more balanced pattern [47]. Transient receptor potential vanilloid 6 and 5 (TRPV6/TRPV5) are highly selective calcium channels that participate in transmembrane Ca^2^⁺ transport in the intestine, kidney and bone. Total glycosides of *C. officinalis* have been shown to directly modulate the TRPV6/TRPV5 pathway [107]. In parallel, serum 17β-oestradiol (E₂) levels increase, suggesting a partial oestrogen-like bone protective effect whereby systemic calcium-phosphate homeostasis is maintained while OVX-induced bone loss is attenuated [107]. Although these findings support a role of *C. officinalis* along the metabolic-bone interaction, the direct molecular targets of its active constituents in metabolic regulation and bone remodelling still require more detailed characterisation.

#### *Crocus sativus* L.

*Crocus sativus* L. (safranal) is a highly valued medicinal spice, widely used as a food additive in the Middle East and employed as a medicinal agent in China. Pharmacological studies have documented antioxidant, anti-inflammatory, antidepressant and chemoprotective properties [28, 104]. Among its constituents, the monoterpene aldehyde safranal has attracted interest in the context of bone resorption. Safranal reduces osteoclast formation and resorption pit area in a dose-dependent manner, and downregulates the expression of receptor activator of RANKL-driven osteoclastogenic markers including NFATc1, CTSK and MMP-9 at both mRNA and protein levels. Sirt1, a nicotinamide adenine dinucleotide (NAD⁺)-dependent deacetylase, is a key regulator of energy metabolism and stress resistance, and its activation is generally associated with metabolic health and cytoprotection [61]. In OVX mice, long-term administration of safranal improves the three-dimensional structure of femoral cancellous bone and upregulates Sirt1 expression, while inhibiting acetylation and NF-κB p65 [61]. These data indicate that saffron, via safranal, activates the Sirt1/NF-κB axis, thereby reducing oestrogen deficiency-related inflammatory and oxidative stress-driven osteoclast overactivity. Given that current conclusions are derived from OVX models within a specific pathophysiological framework, wider validation in additional disease models will be necessary.

#### *Ginkgo biloba* L.

*Ginkgo biloba* L., the leaves of deciduous trees of the Ginkgoaceae family, can be harvested several times during the growing season and has significant resource potential [86, 87]. It has a long history in the application of TCM. It is good at promoting blood circulation and removing blood stasis. It can be used to treat related diseases by improving the blood circulation of the heart and brain, and has the effect of relieving asthma and improving intelligence. The main active ingredients include ginkgo biloba phenols, ginkgo biloba alcohols and other phenolic/alcohol compounds, as well as terpenoid lactone components represented by ginkgolide B (GB). The former has development potential in the fields of health care raw materials, functional foods, pharmaceuticals and cosmeceuticals, while the latter is considered to be an important material basis for anti-oxidation, and anti-inflammation [58, 59, 138].

Related studies have found that GB enhanced ALP activity and mineralized nodules in BMSCs and MC3T3-E1 cells, and promoted the expression of osteogenic genes such as *Runx2*, *Osterix*, *Ocn*, and *Col1*. Furthermore, it activates Wnt/β-catenin pathway by inhibiting glycogen synthase kinase-3β (GSK-3β) and increasing nuclear β-catenin to drive osteogenic differentiation and matrix mineralization [179].

In vivo, GB not only improved trabecular bone structure and BMD in calvaria defect rats and OVX rats, but also significantly increased proximal femur BMD, trabecular volume fraction and cortical bone thickness, while reducing total body fat mass and increasing lean mass in various osteoporosis models such as natural aging, OVX and glucocorticoid-induced osteoporosis, and steady-state adult mice [179]. This suggests that it has both osteogenic assimilative effect and inhibition of bone resorption, accompanied by favorable body composition remodeling. Mechanistically, GB exerts bone protection by regulating oxidative stress, a key metabolic phenotype. In BMSC and osteoblasts from aged mice, GB significantly reduced the levels of ROS and superoxide anion, up-regulated the expression of ROS scavenging enzymes such as Sod2 and catalase, and increased SOD activity. When Sod2/catalase was interfered or knocked down, the promotion effect of GB on osteogenic differentiation and the down-regulation effect on Rankl expression were partially offset, indicating that its promotion and inhibition of osteogenesis were largely dependent on the ROS-related metabolic remodeling process. Consistent with this, GB also reduced the levels of ROS and superoxide anion in aged macrophages and osteoclasts, inhibited RANKL and exogenous H₂O₂ induced osteoclast differentiation, and inhibited bone resorption by increasing the OPG/RANKL ratio, thereby achieving a balance regulation of bone remodeling at the bone microenvironment level with antioxidant regulation and osteoclast inhibition as the core [56].

In addition to the local bone microenvironment, GB also interferes with systemic metabolism and bone remodeling through the "gut-bone" axis. In OVX mice, GB restores the expression of tight junction proteins zonula occludens-1 (ZO-1) and occludin in the colon, reduces TNF-α and IL-6, regulates the ratio of T cell (Treg)/T helper 17 (Th17) and the level of SCFAs, and corrects the imbalance of bacterial flora, indicating that GB regulates bone tissue remodeling through intestinal barrier and low-grade inflammation, and then interferes with estrogen deficience-associated osteoporosis [56]. Notably, Bilobalide, another characteristic component of Ginkgo bilobalide, has also been shown to have anti-osteoporosis potential, but its effect is focused on directly regulating the bone immune microenvironment. Similar to GB, Bilobalide inhibited osteoclast formation and reduced the level of ROS. However, its unique mechanism is that it directly targets and upregulates the deacetylase SIRT3 to inhibit the NF-κB signaling pathway. At the same time, it drives the polarization of macrophages to M2 anti-inflammatory phenotype in a SirT3-dependent manner. In vivo experiments confirmed that OVX mice Bilobalide could significantly improve bone microstructure, accompanied by up-regulation of SIRT3 expression and increase of M2 macrophages in bone tissue [95].

Taken together, the available evidence suggests that the active ingredients in ginkgo biloba, such as GB and bilobalide, can systematically affect the metabolism-bone axis and play an anti-osteoporosis role by regulating multiple metabolic processes such as oxidative stress, inflammation and gut microbiota. However, the efficacy and safety of GB and bilobalidein different osteoporosis phenotypes and clinical populations still need to be further studied.

#### Glycyrrhiza glabra

*Glycyrrhiza* spp. (licorice) is a classical MFH material with a long history of use in Ayurveda, TCM, Persian medicine and European folk practice, where it has been prescribed for joint pain, inflammation and restoration of systemic balance, and is often regarded as a “harmonising” component in multi-herb formulas [93]. Glycyrrhizin (GL) is one of the major triterpenoid saponins in licorice extracts and exhibits broad pharmacological activities, including anti-inflammatory, antiviral and hepatoprotective effects [6]. It can be hydrolysed to the aglycone glycyrrhetinic acid (GA), which exists as two stereoisomers, 18α and 18β [6]. Among these, 18β-glycyrrhetinic acid (18β-GA) is a pentacyclic triterpenoid that has been characterised in bone-related models. In BMMs and RAW264.7 cells, 18β-GA, at concentrations that do not impair precursor cell proliferation or osteogenic and adipogenic differentiation of BMSCs, markedly reduces receptor activator of RANKL-induced tartrate-resistant acid TRAP⁺ multinucleated osteoclast formation and resorption pit area on bone slices. This is accompanied by downregulation of osteoclast-related genes, including *Trap*, *Ctsk*, *Mmp-9* and *Ctr*, and disruption of the F-actin sealing zone structure. Mechanistically, 18β-GA decreases receptor activator of RANK expression and interferes with its interaction with TRAF6, thereby suppressing activation of NF-κB and MAPK pathways, including ERK, JNK and p38, and preventing the downstream upregulation of NFATc1. In OVX mice, 18β-GA attenuates trabecular bone loss, lowers serum TRAP5b, CTX-1, TNF-α and IL-6 and increases mineral apposition rate, indicating that suppression of inflammation-dependent osteoclast activation and inflammatory bone resorption is central to its bone-protective profile [12]. Although licorice has been extensively studied from pharmacological and ethnomedicinal perspectives, additional work in metabolically driven osteoporosis models will be required to clarify how the glycyrrhizin glycyrrhetinic acid axis interfaces with systemic metabolic regulation and skeletal remodeling.

#### *Zingiber officinale* Rosc.

*Zingiber officinale* Rosc. (ginger) is a rhizomatous medicine-food homology plant with several centuries of documented medicinal use. It is traditionally employed to treat diarrhoea, gastrointestinal discomfort, nausea, cholera, asthma and respiratory disorders [2]. Ginger contains phenolic compounds, polysaccharides, volatile oils, terpenoids and organic acids, and has shown beneficial effects in chronic inflammatory and oxidative stress-related conditions [73, 74, 108, 145]. In the food industry, ginger essential oil is widely used as a natural preservative [31]. NADPH oxidase 1 (NOX1), a member of the NADPH oxidase family, is a major source of superoxide and other ROS. The sesquiterpene alcohol cedrol, derived from ginger, markedly reduces osteoclast numbers and resorption lacunae on bovine bone slices, suppresses intracellular ROS accumulation and NOX1 expression and concomitantly increases the levels of antioxidant enzymes such as HO-1 and catalase. At the signalling level, cedrol inhibits activation of NF-κB (reduced phosphorylation and nuclear translocation of p65) and MAPK, including ERK, JNK and p38, and downregulates NFATc1, c-Fos and downstream osteoclast-associated genes such as acid *Acp5*, *Ctsk* and *Atp6v0d*2 [147]. In OVX mice, cedrol improves trabecular bone structure and reduces TRAP^+^ osteoclasts without evident adverse effects on body weight or hepatic and renal function, supporting its potential as a relatively safe anti-resorptive candidate. The co-reported metabolic readouts and osteoprotective outcomes of MFH-derived saponins and terpenoids are summarized in Table 4.

**Table 4:** MFH-derived saponins and terpenoids: co-reported metabolic readouts and osteoprotective outcomes in osteoporosis models

Major category	MFH materials	Active ingredients	Chemical types	OP type	Functions	Mechanisms	References
Saponins	*Astragalus membranaceus* (Fisch.) Bge	Astragaloside IV	Saponins	Primary osteoporosis	Anti-osteoporosis; Anti-oxidation	β-catenin↑, Wnt2↑; FoxO3a↓; MDA↓; CAT↑, SOD↑, GSH-Px↑	[89]
Terpenoids	*Cornus officinalis* Sieb.et Zucc	Total glycosides of Cornus officinalis	Iridoid glycosides	Primary osteoporosis	Promote bone formation; Inhibit bone resorption; Regulate bone metabolism; Estrogen-like activity; Inhibit osteoclastogenesis; Modulate calcium channels	ALP↑, BGP↑; Dpd↓, BMD↑; TRPV6/TRPV5 ratio modulation;	[107]
	*Crocus sativus* L.	Safranal	Monoterpene aldehydes	Primary osteoporosis	Inhibit osteoclastogenesis; Inhibit bone resorption; Prevent bone loss; Anti-inflammatory	Sirt1↑ → p65 acetylation↓ → NF-κB↓; IKK activity↓ → IκBα degradation↓	[109]
	*Angelica sinensis* (Oliv.) Diels	Ligustilide	Phthalides	Secondary osteoporosis	Promote bone formation; Enhance osteoblast differentiation; Anti-oxidation; Anti-apoptosis	GPR30/EGFR–mediated ERK1/2 and Akt activation; Bcl-2↑; H₂O₂-induced ROS and apoptosis↓	[153]
				Primary osteoporosis	Prevent bone loss; Anti-inflammatory; Anti-oxidation	Serum ALP↓, CTx↓, OC↓; BMD↑ (estrogen-independent); IL-1β↓, TNF-α↓	[62]
	*Zingiber officinale* Rosc	Cedrol	Sesquiterpene alcohol	Primary osteoporosis	Inhibit osteoclastogenesis; Inhibit bone resorption; Anti-oxidation	NF-κB↓, MAPK (ERK/JNK/p38)↓; NFATc1 activity↓; ROS↓; HO-1↑, catalase↑	[147]
	*Ginkgo biloba* L.	Bilobalide	Terpene lactones	Primary osteoporosis	Inhibit osteoclastogenesis; Promote M2 macrophage polarization; Improve bone microarchitecture; Anti-oxidation	SIRT3/NF-κB axis modulation; p-p65↓; IκBα↓; ROS↓	[95]
		Ginkgolide B		Primary/secondary	Reverse osteoporosis; Regulate bone homeostasis; Anti-inflammatory; Anti-oxidation	OPG/RANKL ratio↑; ALP↑, Runx2↑; TRAP↓; ROS↓; SOD2↑, catalase↑	[56]
		Ginkgolide B		Primary osteoporosis	Promote osteoblast differentiation; Increase bone formation; Alleviate osteoporosis; Attenuate bone loss via gut–bone axis modulation	BMD↑, BV/TV↑, Tb.Sp↓; CTX-I↓, RANKL↓, PINP↑, OPG↑; Th17/Treg balance modulation; ZO-1↑, occludin↑; gut microbiota (*Lactobacillus*↑) regulation; IL-6↓, TNF-α↓, LPS↓; Wnt/β-catenin↑	[126, 127, 179]
	*Glycyrrhiza glabra*	18β-Glycyrrhetinic acid	Triterpenes	Primary osteoporosis	Inhibit osteoclastogenesis; Inhibit bone resorption; Anti-inflammatory	RANK–TRAF6 interaction↓; NF-κB↓; MAPK↓	[12]

### Proteins and peptides

#### *Dioscorea opposita* Thunb.

*Dioscorea opposita* Thunb. (Chinese yam) is a commonly used MFH rhizome. As discussed above, its steroidal sapogenin diosgenin contributes to the regulation of oestrogen deficiency–related bone loss and lipid abnormalities. Beyond these small molecules, a low-molecular-weight plant protein of approximately 32 kDa, HKUOT-S2, has been isolated from *D. opposita* and shown to possess oestrogen-like activity, adding a protein-level component to its bone-protective profile. In OVX mice, intraperitoneal injection of HKUOT-S2 at 2.18 mg/kg, initiated immediately after surgery and given three times per week for 4 weeks, significantly prevents bone loss in the femur and lumbar spine and improves cortical thickness and other microstructural parameters, with effects comparable to those of 30 μg/kg 17β-oestradiol. In bone tissue and in human mesenchymal stem cells (hMSCs) undergoing osteogenic differentiation, HKUOT-S2 upregulates ERα, ERβ and GPR30, as well as downstream osteogenic markers such as ALP and RUNX2, increases ALP activity and promotes mineralised nodule formation, while having little effect on the number of TRAP-positive osteoclast-like cells. These HKUOT-S2–induced effects are markedly attenuated by the oestrogen receptor antagonist fulvestrant, indicating that HKUOT-S2 delays OVX-related osteoporosis primarily by stimulating local osteoanabolic responses through oestrogen receptors in bone, rather than by directly inhibiting osteoclasts [54]. This protein-level, selectively receptor-mediated oestrogenic mechanism, together with the pseudo-oestrogenic and lipid-regulating actions of diosgenin, delineates a composite pattern by which *D. opposita* engages the metabolic–bone axis.

#### *Gallus gallus domesticus* Brisson

*Gallus gallus domesticus* Brisson (domestic chicken) is widely used as a food animal and is regarded in traditional Chinese medicine as a tonic for “deficiency and fatigue”. Modern studies using proteomics have shown that 83 hepatic proteins are upregulated in *G. gallus domesticus* under immune stress, many of which are associated with oxidative stress and metabolic pathways, indirectly supporting its traditional use in modulating immune function [12]. In addition, genetic characterisation of specific breeds such as the Bian chicken has provided a scientific basis for its commercial development [166, 168, 170]. Collagen extracts derived from *G. gallus domesticus* skeletal muscle (GD collagen) have been shown to improve both bone mass and microarchitecture. In OVX rats, oral administration of GD collagen at 2–3 g/kg for 8 weeks significantly increases cortical and trabecular BMD in the tibia, raises BV/TV, Tb.Th and Tb.N and reduces Tb.Sp. Tibial calcium content and breaking force are also enhanced. At the systemic level, GD collagen partially corrects dyslipidaemia, lowering TC and TG and increasing HDL-C, and attenuates elevated aspartate aminotransferase (AST), suggesting concomitant benefits for hepatic function and lipid metabolism alongside improvements in skeletal outcomes. In human MG-63 osteoblast-like cells, GD collagen promotes ALP activity and mineralised nodule formation, and upregulates the expression of osteogenic markers BMP-2, SMAD family member 5 (SMAD5), Runx2, OCN and COL-1. These changes are accompanied by enhanced phosphorylation of ERK and p38 and suppression of JNK signalling. In RAW264.7 osteoclast precursor cells, GD collagen inhibits receptor activator of RANKL-induced formation of TRAP-positive osteoclasts and resorption pits, decreases *Trap* and *Ctsk* mRNA levels and reduces the RANKL/OPG ratio as well as JNK activation, thereby weakening osteoclast differentiation and resorptive activity [85]. Taken together, collagen derived from *G. gallus domesticus* enhances osteogenesis and suppresses osteoclastogenesis while simultaneously modulating lipid metabolism and hepatic stress, providing a protein-based example of an MFH-derived intervention that targets bone matrix within the broader metabolic–bone framework.

#### *Glycine max* (L.) Merr.

*Glycine max* (L.) Merr. (soybean) provides not only isoflavones and polysaccharides but also storage proteins with skeletal effects. The storage protein β-conglycinin, after phytate removal and deamidation, yields a preparation termed phytate-removed and deamidated soybean β-conglycinin (PrDS), which in postmenopausal osteoporosis models exhibits a bone-protective profile centred on improving mineral absorption and correcting high bone turnover [4]. In OVX rats fed a diet containing 4% PrDS for 8 weeks, the apparent intestinal absorption of Ca^2^⁺ increases from about 38% in OVX controls to values approaching those of sham-operated animals (above 60%), and Mg and Zn absorption rates rise in parallel. Serum parathyroid hormone (PTH) and urinary Dpd, both elevated after OVX, are significantly reduced. These changes are accompanied by significant increases in cortical, trabecular and total femoral BMD, and three-point bending tests show greater fracture resistance with PrDS than with phytate-removed only protein (PrS) or casein. Structural analyses indicate that deamidation increases negative charge on the protein surface, favouring the formation of soluble complexes with Ca^2^⁺ and thereby improving the bioavailability of calcium, magnesium and zinc in the intestine. On this basis, PrDS suppresses secondary hyperparathyroidism and collagen degradation and improves bone microarchitecture and mechanical properties without engaging classical oestrogen receptor pathways or acting as direct hormone replacement. Thus, soybean β-conglycinin–derived proteins can be viewed as an MFH-based protein intervention that approaches the metabolic–bone axis through nutritional modulation of mineral metabolism and opens a new avenue for nutrition-oriented strategies to support skeletal health.

### Saponins

#### *Astragalus membranaceus* (Fisch.) Bge.

Beyond the Astragalus polysaccharides and the isoflavone calycosin described above, studies have also highlighted the triterpenoid saponin astragaloside as an active constituent of *Astragalus membranaceus* (Fisch.) Bge. In OVX rat models, oral administration of astragaloside at 20–80 mg/kg for 8 weeks significantly increases femoral and lumbar spine BMD, improves maximal load and maximal stress in tibial three-point bending tests and elevates serum OPG and calcitonin (CT) levels, while reducing BGP and receptor activator of RANKL. These changes are accompanied by a decrease in MDA and increases in the activities of SOD, GSH-Px and CAT, indicating a shift in bone remodelling from a high-turnover state towards a more balanced pattern under reduced oxidative stress. Mechanistic work has focused on key nodes in oxidative stress signalling. Phosphorylated p66Shc (p-p66Shc) is recognised as an important pro-oxidant adaptor, whereas FoxO3a is a stress-responsive transcription factor that can divert β-catenin away from canonical Wnt signalling [83, 119, 177]. At the protein level, astragaloside downregulates p-p66Shc/p66Shc and FoxO3a, while upregulating Wnt2 and β-catenin expression. These findings suggest that astragaloside attenuates oxidative stress, weakens FoxO3a-mediated competition for β-catenin and restores Wnt2/β-catenin signalling, thereby achieving simultaneous modulation of bone remodelling and metabolic stress in an oxidative stress-driven postmenopausal osteoporosis phenotype [89]. On the basis of these data, it will be important to extend evaluation of astragaloside to additional in vivo settings, such as ageing- and glucocorticoid-induced osteoporosis models, in order to clarify how generalisable its metabolic-bone actions are and to dissect potential similarities and differences in its mechanisms across distinct aetiological backgrounds. The corresponding evidence for MFH-derived saponins is also presented in Table 4.

### Other specialized metabolites

#### *Cannabis sativa* L.

*Cannabis sativa* L. is a traditional oilseed crop with MFH characteristics and multiple uses, including medicinal preparations, edible seed oil and fibre. Genetic studies have revealed substantial variation in photoperiod sensitivity [16], and analysis of the genetic and phytochemical diversity of 61 *C. sativa* accessions (the parental line CIM-CS-64 and 60 half-sib progeny) suggests considerable potential for soil nutrient restoration in agricultural systems [3]. The non-psychoactive phytocannabinoid cannabidiol (CBD) has recently attracted interest in bone biology. In human osteoprogenitor and osteoblast precursor cultures, 1–10 μg/mL CBD increases cell viability and proliferation and upregulates osteogenic markers such as osteocalcin, without detectable cytotoxicity. In vivo, CBD has been evaluated in two osteoporosis models: fluoxetine-induced drug-related bone loss and OVX-induced osteoporosis in female mice. Continuous subcutaneous infusion of CBD at 5 mg/kg prevents the decline in BMD and deterioration of trabecular microarchitecture in both models, increasing BV/TV, Tb.Th and Tb.N and reducing Tb.Sp. In OVX animals, CBD also improves delayed femoral fracture healing, with callus quality approaching that of non-OVX controls [39]. In addition to these skeletal endpoints, an extended OVX study reported that oral CBD was accompanied by improved glucose tolerance and higher oxygen consumption and energy expenditure, together with lower colonic Il1b, Il6 and Tnf expression and higher Tjp1 expression, consistent with attenuation of intestinal inflammatory tone and support of barrier related programs. Although cecal SCFAs were not detectably altered, bile acid profiles and 16S based analyses indicated microbiota restructuring that included *Lactobacillus* enrichment, and these systemic and intestinal changes coincided with reduced femoral Tnfrsf11 expression and attenuation of OVX associated Il6 upregulation [114]. Collectively, current evidence supports CBD as a candidate MFH related bioactive with concurrent skeletal and metabolic correlates, while the causal contribution of microbiota and bile acid changes to bone protection remains to be resolved.

#### *Codonopsis pilosula* (Franch.) Nannf.

*Codonopsis pilosula* (Franch.) Nannf. is an MFH root used in traditional Chinese medicine to tonify qi, strengthen the spleen and lung and nourish blood and body fluids [75]. Polysaccharides from *C. pilosula* (Codonopsis pilosula polysaccharides, CPPs) are recognised as major bioactive components with immunomodulatory, antioxidant, hypoglycaemic and gut microbiota–modulating effects [67, 69, 86, 87, 126, 127]. The polyacetylene lobetyolin represents a low-molecular-weight constituent with direct effects on bone remodelling. At concentrations that do not affect the viability of BMMs or mesenchymal stromal cells, lobetyolin suppresses osteoclast formation and resorption pit development, downregulates osteoclast-related genes including Trap, Ctsk, Nfatc1 and c-Fos and reduces ROS generation. In LPS-induced models of impaired osteogenesis, lobetyolin improves ALP activity and mineralised nodule formation in BMSCs, partially restoring their osteogenic potential. Mechanistic data indicate that lobetyolin blocks nuclear translocation of NF-κB p50/p65 and inhibits the downstream NFATc1/c-Fos axis, thereby weakening inflammation-driven osteoclast activation. In OVX mice, intraperitoneal lobetyolin at 20 mg/kg for 5 weeks improves trabecular microarchitecture and reduces TRAP-positive osteoclasts and resorption surfaces. Overall, the available evidence supports a dual profile in which lobetyolin attenuates inflammatory and oxidative signalling, limits osteoclastogenesis and preserves osteoblast function, providing a concrete molecular basis for the contribution of *C. pilosula* to correcting metabolic and inflammatory stress–induced disturbances of bone remodelling along the metabolic–bone axis [146]. The corresponding evidence for MFH-derived proteins & peptides and other specialized metabolites is summarized in Table 5.

**Table 5:** MFH-derived proteins/peptides and other specialised metabolites: co-reported metabolic readouts and osteoprotective outcomes in osteoporosis models

Major category	MFH materials	Active ingredients	Chemical types	OP type	Functions	Mechanisms	References
Proteins & Peptides	*Gallus gallus domesticus* Brisson	Collagen	Collagens	Primary osteoporosis	Increase bone strength, calcium content, BMD and microarchitecture; Promote osteogenesis; Inhibit osteoclastogenesis; Improve lipid metabolism	BMP-2/SMAD5/Runx2 and Wnt signaling↑; OPG↑, RANKL↓; JNK↓, p38↑; TC↓, HDL↑	[85]
	*Dioscorea opposita* Thunb	HKUOT-S2 protein	Proteins	Primary osteoporosis	Anti-osteoporosis; Promote osteogenic differentiation	ERα↑, ERβ↑, GPR30↑; ALP↑, RUNX2↑, COL1A1↑	[54]
	*Glycine max* (L.) Merr	Phytate-removed and deamidated soybean proteins	Proteins	Primary osteoporosis	Enhance calcium absorption; Inhibit bone resorption; Increase BMD and bone strength	PTH↓, DPD↓; bone mineral density↑, bending strength↑; Ca/Mg/Zn absorption↑	[4]
Other specialized metabolites	*Cannabis sativa* L.	Cannabidiol	Cannabinoids	Primary/secondary	Prevent osteoporosis; Enhance osteoblast activity; Anti-inflammatory	Runx2↑, osteocalcin gene expression↑; cell viability/proliferation↑; BMD loss↓	[39]
	*Codonopsis pilosula* (Franch.) Nannf	Lobetyolin	Polyacetylenes	Primary osteoporosis	Inhibit osteoclastogenesis; Promote osteogenic differentiation; Anti-inflammatory; Anti-oxidation	NF-κB p65/p50 nuclear translocation↓; NFATc1↓, c-Fos↓; ROS↓	[146]

## Discussion

Osteoporosis is characterized by progressive loss of bone mass and deterioration of bone microstructure, putting the risk of fragility fractures at increased risk. With the deepening of medical research, bone loss is not only caused by local changes in the function of osteoblasts and osteoclasts, but also by systemic changes in lipid metabolism, inflammatory activation, REDOX homeostasis, and inter-organ signaling. It is gradually regarded as a comprehensive result of functional imbalance of multiple organs. In this structured evidence synthesis, 38 bioactive components from 24 drug-food homologues were identified as important for the regulation between metabolism and bone. Although the included MFH bioactives were classified according to chemical features, several class-related mechanistic tendencies can still be observed. Flavonoids and isoflavones were frequently associated with regulation of osteoblast–osteoclast balance, involving Wnt/β-catenin activation, RANKL/NFATc1 modulation, and improvement of lipid or inflammatory status. Polysaccharides were more often linked to gut microbiota remodeling, SCFA production, intestinal barrier protection, calcium absorption, and anti-inflammatory regulation. Phenolic and polyphenolic compounds mainly converged on antioxidant and anti-inflammatory mechanisms, including ROS suppression, Nrf2/HO-1 activation, NF-κB inhibition, and reduced osteoclastogenesis. These observations suggest that different MFH chemical classes may show partially distinct mechanistic tendencies while ultimately converging on the restoration of bone remodeling balance. It is also noteworthy that improvements in osteoporosis-related phenotypes were frequently accompanied by parallel changes in systemic metabolic status, inflammatory tone, oxidative stress, or gut-derived signals. These observations indicate that, in metabolically disturbed settings, the actions of MFH bioactives may extend beyond bone-local regulation alone. Although direct causal links remain to be validated, the available evidence provides a rationale for interpreting part of their osteoprotective effects from an organ-bone axis perspective. Accordingly, the following sections discuss the potential trans-organ regulatory patterns of MFH bioactives through the liver-bone and gut-bone axis. It should be noted that the liver-bone and gut-bone axes are discussed here as plausible interpretive frameworks rather than fully established causal pathways. Because many included studies reported parallel changes in bone, metabolic, hepatic, microbial, or inflammatory indicators, these findings should be interpreted as indirect mechanistic inference rather than definitive proof of causality.

### The liver-bone axis: Bone effects mediated by liver metabolites

In the study of active ingredients from MFH included in this research, the liver-bone axis is mainly characterized by the simultaneous improvement of bone phenotypes and lipid metabolism-related indicators or metabolomic features. For example, in the OVX model, daidzin and glycitin improve bone mineral density while alleviating the increase in total cholesterol and triglycerides [125]. WSSF not only improves the hypercholesterolemia phenotype but also reports improved bone outcomes accompanied by increased intestinal calcium absorption [84]. In the metabolomics-guided study of puerarin, the improvement of trabecular bone structure is parallel to changes in blood lipids, accompanied by the remodeling of pathways related to phospholipids and polyunsaturated fatty acids and changes in PPARγ. In the type 2 diabetes model, puerarin can also alleviate bone loss and bone marrow adiposity aggravated by pioglitazone, and at the same time, changes in glycerophospholipid and α-linolenic acid metabolism are observed. This parallel improvement provides a clear entry point: when explaining the bone-protective effect of MFH, changes in liver metabolic status and its possible systemic signal input should be considered simultaneously, rather than being limited to local bone pathways. On this basis, the liver-bone axis can be organized into three interrelated signaling layers: lipid transport and cholesterol homeostasis represented by LCAT, anabolic endocrine support represented by IGF-1, and metabolic stress-related hepatokines represented by the FGF21/IGFBP1 pathway.

From the perspective of the liver-bone axis, changes at the liver end during metabolic abnormalities mainly include three types: decreased lipid transport ability, weakened anabolic signals for osteogenesis, and upregulation of liver factors related to metabolic stress that promote bone resorption. The liver is the main site of lipid metabolism. LCAT is the only enzyme in plasma that can esterify cholesterol and promote the esterification of cholesterol with phospholipid-derived acyl chains [156, 158]. Under pathological conditions, the increase of PP2Acα in liver tissue inhibits the transcriptional expression of LCAT in hepatocytes by dephosphorylating USF1, resulting in a decrease in serum LCAT, thereby weakening the ability of HDL cholesterol esterification and reverse cholesterol transport, making it more likely to stay in peripheral cells and causing abnormal lipid metabolism [73, 74]. This change in the local lipid metabolic environment, especially the imbalance of cholesterol homeostasis around osteoclast precursor cells (derived from the bone marrow monocyte lineage), will directly affect their differentiation and function. Therefore, the decrease in LCAT-mediated reverse cholesterol transport ability may favor bone resorption by changing the lipid metabolic environment of osteoclast precursors [73, 74]. Clinical evidence also provides consistent support: a multi-center cohort study in 2025 showed that serum LCAT in patients with primary osteoporosis was significantly reduced and was positively correlated with T/Z-score, suggesting a positive correlation between serum LCAT and bone mass [134]. In addition to affecting osteoclasts, the impairment of liver metabolic homeostasis can also weaken osteoblast activity, mainly manifested as the weakening of IGF-1 signaling. When liver function is impaired, the level of liver-derived IGF-1 in the circulation decreases, resulting in blocked IGF1R signal transduction in bone tissue [20]. Mechanistically, after IGF-1 binds to IGF1R to form a ligand-receptor complex, it triggers the autophosphorylation of tyrosine in the receptor β subunit, then recruits and phosphorylates IRS-1/2, activates the PI3K-Akt pathway and further drives the mTORC1 pathway, promoting the differentiation of mesenchymal stem cells into the osteogenic lineage and enhancing the anabolic and mineralization processes of osteoblasts. This pathway is considered to be the key molecular basis for IGF-1 to support bone formation and maintain bone mass [141, 180]. Consistently, at the cellular level, IGF-1 can increase ALP activity and enhance the formation of mineralized nodules, suggesting its direct pro-differentiation effect on the osteogenic phenotype [151]. Finally, in a state of metabolic disorder, the liver also secretes FGF21, a typical stress-induced liver factor, which can induce the upregulation of liver IGFBP1 expression and its secretion into the blood. Circulating IGFBP1 binds to integrin β1 on the surface of osteoclast precursor cells through its RGD motif, enhancing the RANKL-mediated ERK signal and NFATc1 transcription program, thereby promoting osteoclast differentiation and bone resorption. At the same time, IGFBP1 blocking antibodies can inhibit this pro-osteoclastic effect [128].

**Fig. 3:**
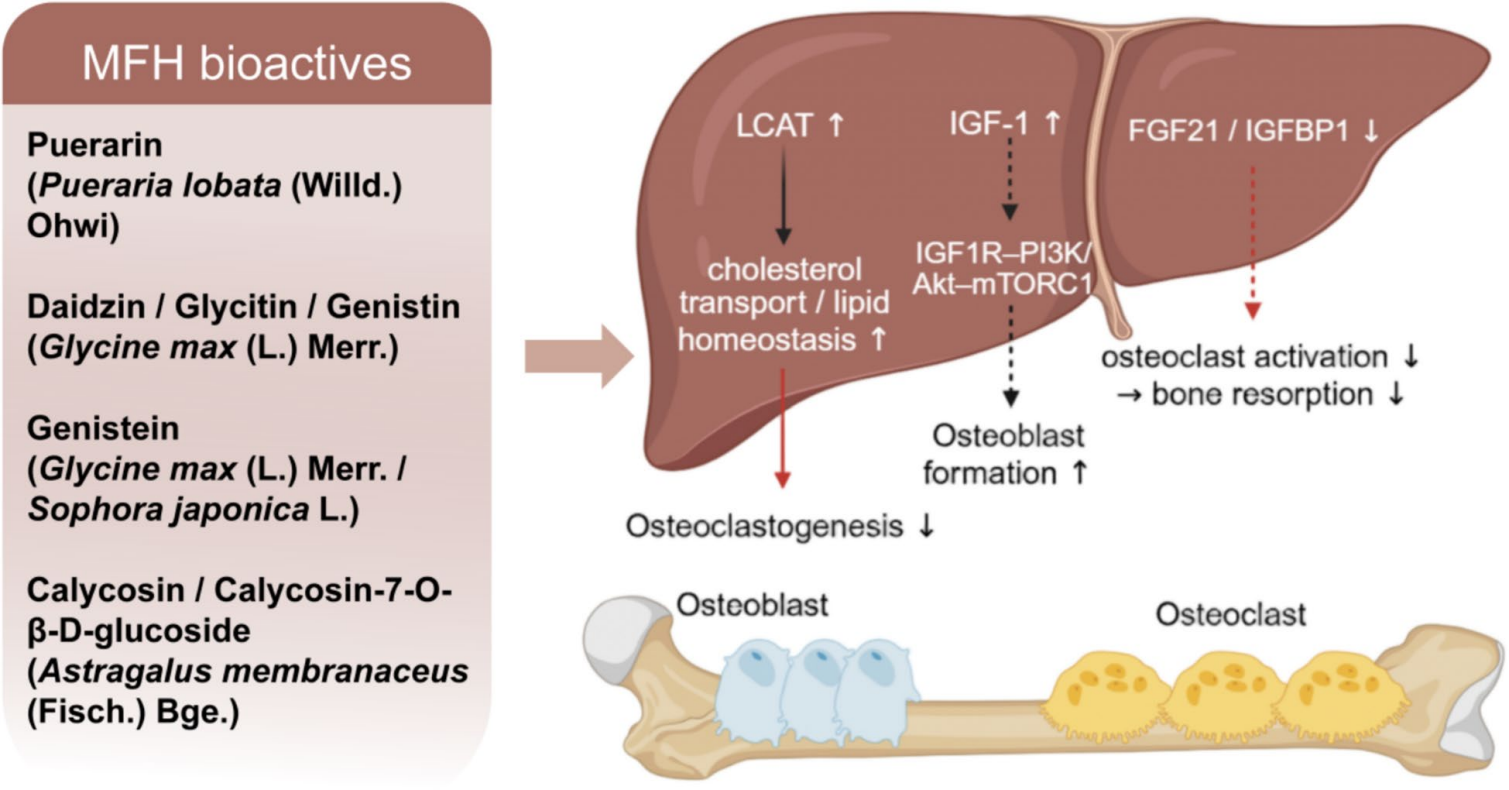
MFH active ingredients ameliorate osteoporosis through the liver-bone axis

In summary, existing evidence suggests that the improvement of the bone microenvironment after intervention with MFH active ingredients often occurs in parallel with the improvement of lipid metabolism-related phenotypes, and the literature supports that multiple liver-derived metabolic or endocrine factors may be involved in liver-bone communication. Therefore, the liver-bone axis should be regarded in this review as a plausible interpretive framework rather than a universally proven causal pathway for MFH-derived bioactives. Current evidence suggests that some MFH interventions improve bone phenotypes in parallel with changes in lipid metabolism and liver-derived metabolic or endocrine signals. However, the current MFH-related evidence for these liver-derived signals remains indirect and uneven. Most studies report concurrent changes in lipid metabolism, metabolomic pathways, or bone phenotypes, whereas direct validation that LCAT, IGF-1, FGF21/IGFBP1, or related lipid mediators are necessary for MFH-induced bone protection is still lacking (Fig. 3).

### The gut-bone axis: regulation of bone homeostasis mediated by gut microbiota and their metabolites

While the bone microstructure is improved, consistent changes also occur in the gut microbiota and the gut barrier. Taking WSSF as an example, in the OVX model, the improvement of osteoporosis is observed simultaneously with an increase in cecal SCFAs and enhanced intestinal calcium absorption, suggesting that changes in microbial metabolism may be involved in the improvement of osteoporosis [84]. In the study of puerarin, the improvement of trabecular bone structure is parallel to the remodeling of the microbiota composition, the increase in SCFAs, and the repair of the gut barrier integrity. In addition, GB intervention upregulates colonic tight junction proteins (ZO-1/occludin), reduces inflammatory factors, and is accompanied by an increase in SCFAs and a decrease in the Th17/Treg ratio. Overall, MFH may regulate gut microbial metabolites and the barrier-inflammatory state through the gut-bone axis to achieve the effect of improving the bone microenvironment. Different MFH-derived bioactives appear to affect this axis through partially distinct gut-derived outputs: polysaccharides and dietary-fiber-like components are more often associated with SCFA production, calcium absorption, and barrier repair; flavonoids and isoflavones are more frequently linked to microbiota remodeling and inflammatory regulation; whereas evidence directly connecting MFH interventions to bile acid-mediated bone effects remains relatively limited.

From a mechanistic perspective, the indirect regulation of bone homeostasis by the gut microbiota does not rely on a single mediator but is mainly achieved through three types of gut-derived output signals: short-chain fatty acids produced by microbial fermentation, secondary bile acid metabolites formed by the microbiota's participation in bile acid conversion, and bacterial-derived inflammatory molecules such as lipopolysaccharide (LPS) that overflow into the blood after gut barrier damage. First, SCFAs produced by gut microbiota fermentation mainly inhibit osteoclastogenesis. Existing studies have shown that propionic acid and butyric acid can inhibit the differentiation of osteoclast precursors into mature osteoclasts and increase bone mass in vivo, reducing postmenopausal and inflammation-related bone loss [76]. At the molecular level, this effect is related to the inhibition of osteoclast-related transcription programs, manifested as the downregulation of key nodes such as TRAF6, c-Fos, and NFATc1, thereby reducing the formation of TRAP⁺ multinucleated osteoclasts and bone resorption activity [51, 76]. Further, the effect of butyric acid cannot be fully explained by a single receptor pathway: early studies have shown that sodium butyrate and other HDAC inhibitors can directly inhibit RANKL-induced osteoclast differentiation [99], while subsequent studies suggest that butyric acid can also participate in its anti-osteoclastic effect by inhibiting HDAC2 in osteoclasts [50]. At the same time, the study by Lucas et al. suggests that the inhibition of osteoclasts by SCFAs is not mainly dependent on GPR41/GPR43 signaling but is closely related to the remodeling of the metabolic program in the early stage of osteoclast precursor differentiation, specifically manifested as enhanced glycolysis, an increased ECAR/OCR ratio, and AMPK activation, accompanied by the downregulation of key osteoclast genes such as TRAF6 and NFATc1 [76]. Therefore, a more reasonable understanding is that gut-derived SCFAs may inhibit osteoclast differentiation through multiple mechanisms other than receptor-dependent signaling, including epigenetic regulation related to HDAC inhibition and cellular metabolic remodeling, thus participating in the indirect regulation of bone homeostasis by the gut-bone axis.

In addition to the microbial fermentation product pathway represented by SCFAs, metabolites produced by bile acid biotransformation may also constitute another important class of gut-derived intermediate signals. HDCA is a secondary bile acid, and its in vivo production is generally considered to be mainly derived from the gut microbiota-mediated bile acid biotransformation [137]. A recent study further proposed that the GM–HDCA–TGR5 axis may be another key mechanism pathway connecting the gut microbiota and bone remodeling: HDCA activates the TGR5 signal, weakens NF-κB (p65) activation, and promotes the polarization of macrophages to the M2 phenotype, thereby reducing osteoclastogenesis and improving the bone microstructure at the animal level [110, 174]. Finally, increased gut permeability can increase the entry of bacterial-derived LPS into the blood, thereby converting gut barrier damage into bone-end inflammatory signals. Existing studies have shown that LPS activates the TLR4/MyD88/NF-κB cascade, on the one hand, inducing the release of inflammatory factors such as TNF-α and IL-1β, and on the other hand, promoting the expression of RANKL in bone-related stromal cells/osteoblasts, ultimately enhancing osteoclast differentiation and bone resorption. However, this effect does not occur completely unconditionally but is closely related to the differentiation stage of osteoclast precursors and is more likely to show a pro-osteoclastic effect after RANKL pre-stimulation [5, 24, 42, 70, 105, 120].

**Fig. 4:**
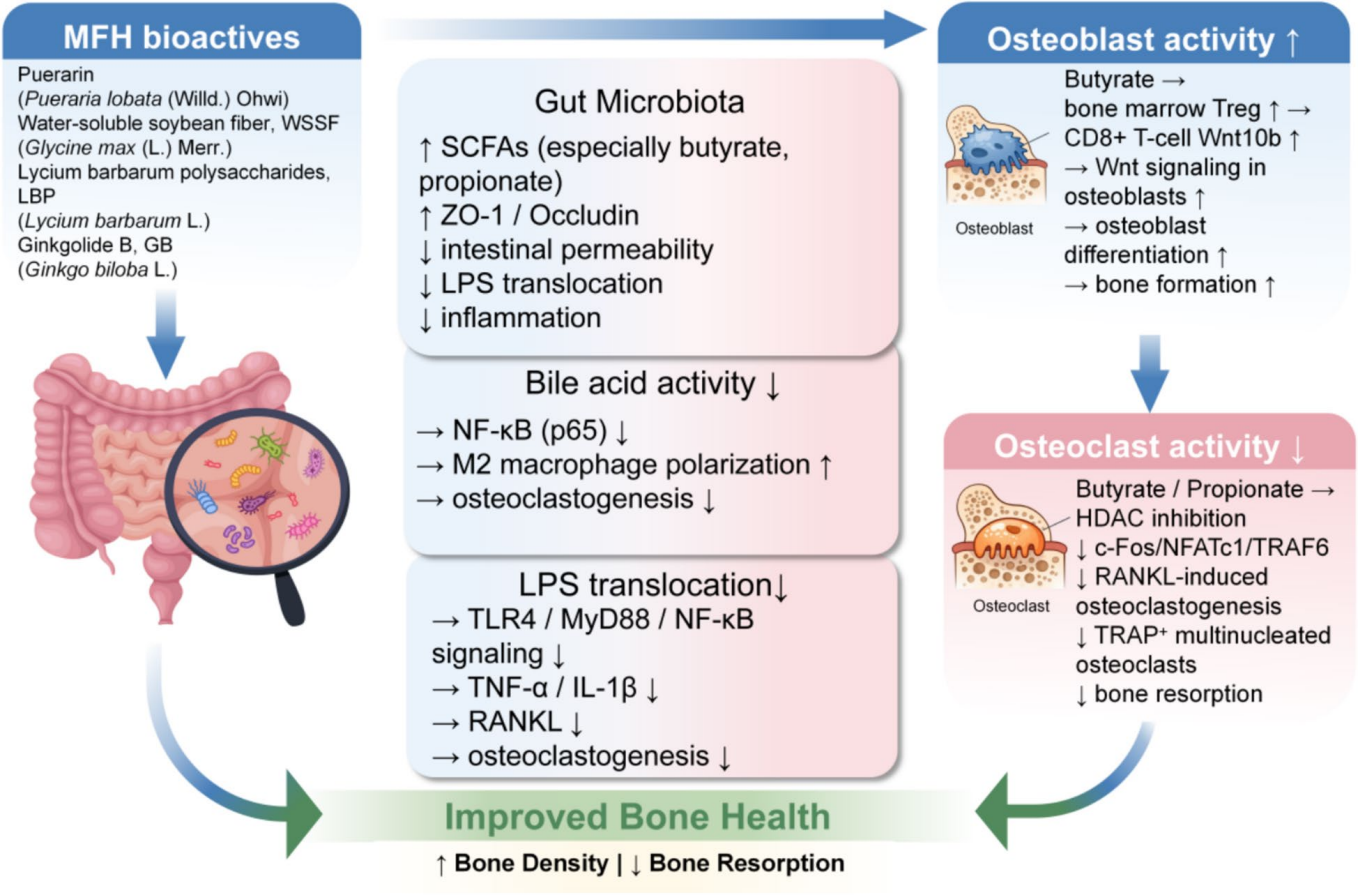
MFH active ingredients ameliorate osteoporosis through the gut-bone axis

In summary, improvements in bone phenotypes after MFH-derived bioactive intervention are often accompanied by gut microbiota remodeling, restoration of barrier function, changes in SCFA or bile acid-related metabolites, and reduction of inflammatory signals. These findings support the gut-bone axis as a plausible explanatory framework for indirect regulation of bone homeostasis. However, the necessity and relative contribution of SCFAs, bile acid-derived metabolites such as HDCA, LPS-related inflammatory signals, and other microbiota-derived mediators remain insufficiently validated across different MFH chemical classes. Future studies using metabolite intervention, microbiota transplantation, antibiotic or germ-free models, receptor blockade, and pathway-specific genetic approaches are needed to determine whether these gut-derived signals are true causal mediators of MFH-induced bone protection (Fig. 4).

### Limitations and future directions

In summary, the findings discussed above can be interpreted through three complementary trans-organ perspectives: liver-bone and gut-bone. This framework helps explain why, under metabolically disturbed conditions, improvements in bone phenotypes are often accompanied by parallel changes in lipid metabolism, gut-derived signals, inflammatory status, and other systemic processes. However, the specific mediators and causal links remain to be established.

Current evidence is still mainly derived from animal and cell studies, and heterogeneity in models, interventions, and outcome measures limits cross-study comparability. A further translational challenge lies in the gap between experimental dosing and realistic human intake. Many studies used purified compounds, standardized extracts, or enriched fractions at doses that may exceed those achievable through routine consumption of the corresponding MFH materials. In addition, extraction procedures, compound purity, food matrix effects, intestinal absorption, metabolism, and bioavailability may substantially influence systemic exposure and biological activity. Therefore, the osteoprotective effects observed in preclinical models should not be directly extrapolated to routine dietary intake or clinical efficacy without pharmacokinetic, dose–response, safety, and human intervention evidence. Moreover, although many studies reported concurrent improvements in bone phenotypes and systemic metabolic or inflammatory parameters, the causal role of these intermediate changes remains insufficiently validated. From the perspective of indirect pharmacology, future studies should clarify whether liver-derived mediators, such as LCAT, IGF-1, FGF21/IGFBP1, lipid metabolites, and bile acid-related signals, or gut-derived mediators, such as SCFAs, HDCA, LPS-related inflammatory signals, and microbiota-derived metabolites, are mechanistically involved in MFH-induced bone protection. This will require integration of multi-omics screening with targeted validation approaches, including metabolite intervention, receptor blockade, microbiota transplantation, antibiotic or germ-free models, organ-specific pathway modulation, and genetic perturbation [73, 74, 76, 123, 128]. Such studies will help determine whether these systemic changes are true mediators of bone protection or merely accompanying responses.

## Conclusion

In summary, the currently included evidence indicates that 38 active ingredients derived from 24 MFH materials can improve osteoporosis-related phenotypes, often alongside parallel changes in metabolism- or gut-related indicators. These findings support the use of a trans-organ indirect regulatory perspective to interpret the actions of MFH bioactives under metabolic dysregulation, while the specific mediators and causal links remain to be clarified.

## Supplementary Information

Supplementary material 1.

## Data Availability

Data will be made available on request.

## Abbreviations

MFH: medicine-food homology
TCM: traditional Chinese medicine
DOP: diabetic osteoporosis
PMOP: postmenopausal osteoporosis
OP: osteoporosis
OVX: ovariectomy / ovariectomized
BMD: bone mineral density
BMC: bone mineral content
BMSCs: bone marrow mesenchymal stem cells
BMDMs: bone marrow-derived macrophages
BMMs: bone marrow macrophages
HFD: high-fat diet
STZ: streptozotocin
LTG: liquiritigenin
APS: Astragalus polysaccharide
PSP: Polygonatum sibiricum polysaccharide
PCP: Polygonatum cyrtonema polysaccharide
LBP: Lycium barbarum polysaccharide
LBP-GP: Lycium barbarum polysaccharide-glycoprotein complexes
SCFAs: short-chain fatty acids
ALP: alkaline phosphatase
BALP: bone-specific alkaline phosphatase
BGP: bone Gla protein
BMP: bone morphogenetic protein
BMP4: bone morphogenetic protein 4
bFGF: basic fibroblast growth factor
CRP: C-reactive protein
ER-α: estrogen receptor α
IL-1β: interleukin-1β
PINP: procollagen type I N-terminal propeptide
PICP: procollagen type I C-terminal propeptide
RANKL: receptor activator of nuclear factor kappa-B ligand
Runx2: runt-related transcription factor 2
TRAP / TRACP-5b: tartrate-resistant acid phosphatase / tartrate-resistant acid phosphatase 5b
8-OHdG: 8-hydroxy-2'-deoxyguanosine
CAT: catalase
FoxO3a: forkhead box O3a
ROS: reactive oxygen species
MDA: malondialdehyde
SOD: superoxide dismutase
GSH-Px: glutathione peroxidase
IL-6: interleukin-6
TNF-α: tumor necrosis factor-α
TGF-β: transforming growth factor-β
TGF-βR2: transforming growth factor-β receptor 2
TNFSF10: TNF superfamily member 10
MMP-13: matrix metalloproteinase-13
MSX2: Msh homeobox 2
NFATc1: nuclear factor of activated T cells cytoplasmic 1
JNK: c-Jun N-terminal kinase
PI3K/Akt: phosphoinositide 3-kinase/protein kinase B
mTOR: mechanistic target of rapamycin
NF-κB: nuclear factor kappa B
Nrf2/HO-1: nuclear factor erythroid 2-related factor 2/heme oxygenase-1
NO/cGMP: nitric oxide/cyclic guanosine monophosphate
NQO1: NAD(P)H quinone dehydrogenase 1
STING: stimulator of interferon genes
Trx1: thioredoxin 1
TrxR1: thioredoxin reductase 1
VEGF: vascular endothelial growth factor
