# Supplementary material for: Medicine–food homologous bioactives in metabolic dysregulation-associated osteoporosis: a review of preclinical evidence and potential liver–bone and gut–bone actions
Source: Chin Med. 2026 Jul 22;21:201. doi: 10.1186/s13020-026-01453-6 (PMC13390145; doi:10.1186/s13020-026-01453-6)
Supplement: Supplementary file 1 — Supplementary material 1. [file 13020_2026_1453_MOESM1_ESM.docx]

**Supplementary table**

**Table S1. Medicinal and food homologous (MFH) species reported to exert metabolic regulatory and anti-osteoporotic effects.**

| **No.** | **MFH morphology** | **MFH Latin name** | **English common name** | **Name of department** | Part of use | Metabolic regulation evidence  （Y/N） | **anti-osteoporotic evidence**  （Y/N） |
| --- | --- | --- | --- | --- | --- | --- | --- |
| 1 | 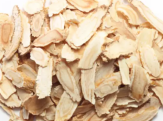 | Angelica sinensis (Oliv.) Diels. | dong quai (Chinese angelica root) | Umbelliferae | root | Y | Y |
| 2 | 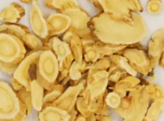 | Astragalus membranaceus (Fisch.) Bge. | astragalus root (huangqi) | Leguminosae | root | Y | Y |
| 3 | 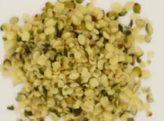 | Cannabis sativa L. | hemp seed | Sanko | mature fruit | Y | Y |
| 4 | 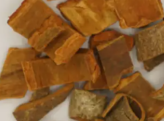 | Cinnamomum cassia Presl | Chinese cinnamon (cassia) | Lauraceae | bark | Y | Y |
| 5 | 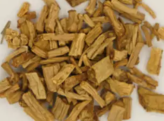 | Codonopsis pilosula (Franch.) Nannf. | codonopsis root (dangshen) | Campanulaceae | root | Y | Y |
| 6 | 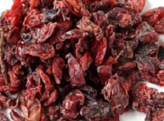 | Cornus officinalis Sieb. et Zucc. | Asiatic cornelian cherry fruit | Cornaceae | fruit | Y | Y |
| 7 | 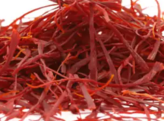 | Crocus sativus L. | saffron stigma | Iridaceae | stigma | Y | Y |
| 8 | 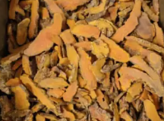 | Curcuma longa L. | turmeric rhizome | Zingiberaceae | rhizome | Y | Y |
| 9 | 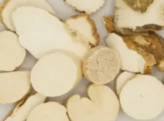 | Dioscorea opposita Thunb. | Chinese yam | Dioscoreaceae | rhizome | Y | Y |
| 10 | 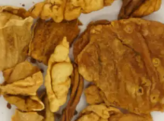 | Gallus gallus domesticus Brisson | chicken gizzard lining | Phasianidae | inner lining of the gizzard | Y | Y |
| 11 | 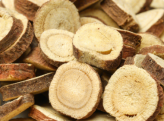 | *Glycyrrhiza glabra* | licorice | Fabaceae | root and rhizome | Y | Y |
| 12 | 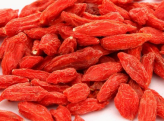 | Lycium barbarum L. | goji berry (wolfberry) | Solanaceae | ripe fruit | Y | Y |
| 13 | 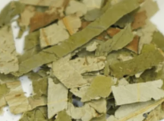 | Nelumbo nucifera Gaertn. | lotus leaf | Nymphaeaceae | leaf | Y | Y |
| 14 | 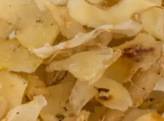 | Polygonatum cyrtonema Hua | Solomon’s seal rhizome | Liliaceae | rhizome | Y | Y |
| 15 | 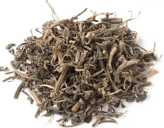 | Portulaca oleracea L. | purslane | Portulacaceae | aerial parts | Y | Y |
| 16 | 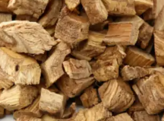 | Pueraria lobata (Willd.) Ohwi | kudzu root | Fabaceae | root | Y | Y |
| 17 | 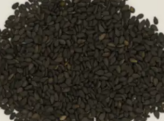 | Sesamum indicum L. | black sesame seed | Pedaliaceae | ripe seeds | Y | Y |
| 18 | 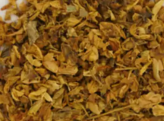 | Sophora japonica L. | pagoda tree flower (Sophora flower) | Fabaceae | flower | Y | Y |
| 19 | 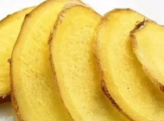 | Zingiber officinale Rosc. | ginger rhizome | Zingiberaceae | rhizome | Y | Y |
| 20 | 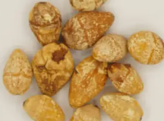 | Ginkgo biloba L. | ginkgo nut | Ginkgoaceae | ripe seeds | Y | Y |
| 21 | 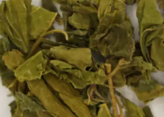 | Morus alba L. | mulberry leaf | Moraceae | leaf | Y | Y |
| 22 | 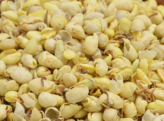 | *Glycine max* (L.) Merr. | fermented soybean (douchi) | Fabaceae | fermented product of ripe seeds | Y | Y |
| 23 | 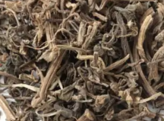 | *Polygonatum sibiricum* Red. | Solomon’s seal rhizome | Liliaceae | rhizome | Y | Y |
| 24 | 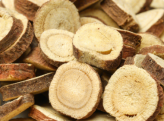 | Glycyrrhiza uralensis Fisch. | licorice | Fabaceae | root and rhizome | Y | Y |

**Table S2. MFH plants, active ingredients, chemical types and structural information.**

| **No.** | **MFH material** | **Active ingredients** | **Major category** | **Chemical types** | **Structural information** | **PubChem CID** | **SMILES** |
| --- | --- | --- | --- | --- | --- | --- | --- |
| 1 | Curcuma longa L. | Curcumin | Phenolic and polyphenolic compounds | Polyphenols | 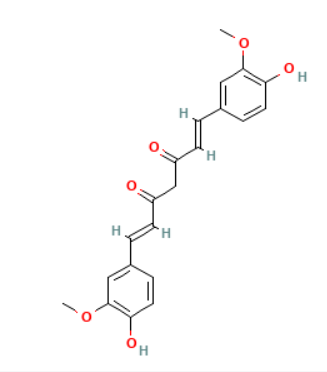 | 969516 | COC1=C(C=CC(=C1)/C=C/C(=O)CC(=O)/C=C/C2=CC(=C(C=C2)O)OC)O |
| 2 | Morus alba L. | Mulberroside A | Phenolic and polyphenolic compounds | Stilbenoid glycosides | 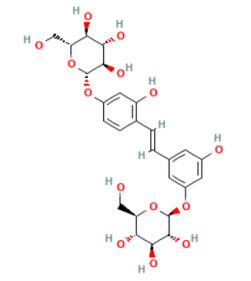 | 6443484 | C1=CC(=C(C=C1O[C@H]2[C@@H]([C@H]([C@@H]([C@H](O2)CO)O)O)O)O)/C=C/C3=CC(=CC(=C3)O[C@H]4[C@@H]([C@H]([C@@H]([C@H](O4)CO)O)O)O)O |
| 3 | Morus alba L. | Chlorogenic acid | Phenolic and polyphenolic compounds | Tannins | 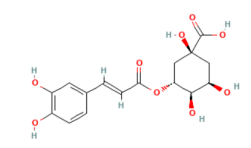 | 1794427 | C1[C@H]([C@H]([C@@H](C[C@@]1(C(=O)O)O)OC(=O)/C=C/C2=CC(=C(C=C2)O)O)O)O |
| 4 | Morus alba L. | Morusinol | Flavonoids | Flavonoids | 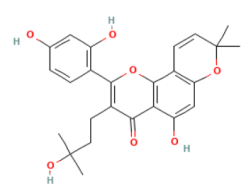 | 5481968 | CC1(C=CC2=C(O1)C=C(C3=C2OC(=C(C3=O)CCC(C)(C)O)C4=C(C=C(C=C4)O)O)O)C |
| 5 | Cinnamomum cassia Presl | Corilagin | Phenolic and polyphenolic compounds | Tannins | 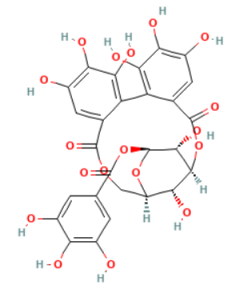 | 73568 | C1[C@@H]2[C@H]([C@@H]([C@H]([C@@H](O2)OC(=O)C3=CC(=C(C(=C3)O)O)O)O)OC(=O)C4=CC(=C(C(=C4C5=C(C(=C(C=C5C(=O)O1)O)O)O)O)O)O)O |
| 6 | Sesamum indicum L. | Sesamin | Phenolic and polyphenolic compounds | Lignans | 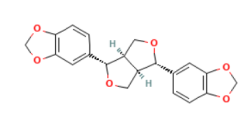 | 72307 | C1[C@H]2[C@H](CO[C@@H]2C3=CC4=C(C=C3)OCO4)[C@H](O1)C5=CC6=C(C=C5)OCO6 |
| 7 | Angelica sinensis (Oliv.) Diels. | Guaiacol | Phenolic and polyphenolic compounds | Phenols | 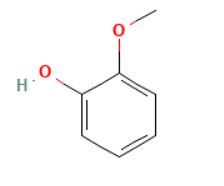 | 460 | COC1=CC=CC=C1O |
| 8 | Angelica sinensis (Oliv.) Diels. | Ligustilide | Terpenoids | Phthalides | 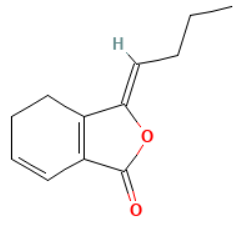 | 5319022 | CCC/C=C\1/C2=C(C=CCC2)C(=O)O1 |
| 9 | *Glycyrrhiza glabra* | Liquiritigenin | Flavonoids | Flavonoids | 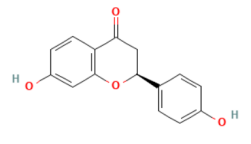 | 114829 | C1[C@H](OC2=C(C1=O)C=CC(=C2)O)C3=CC=C(C=C3)O |
| 10 | *Glycyrrhiza glabra* | 18β-Glycyrrhetinic acid | Terpenoids | Triterpenes | None | None | None |
| 11 | Glycyrrhiza uralensis Fisch. | Liquiritigenin | Flavonoids | Flavonoids | 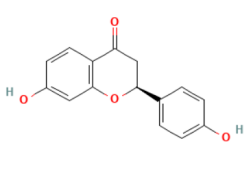 | 114829 | C1[C@H](OC2=C(C1=O)C=CC(=C2)O)C3=CC=C(C=C3)O |
| 12 | Sophora japonica L. | Sophoricoside | Flavonoids | Isoflavone glycosides | 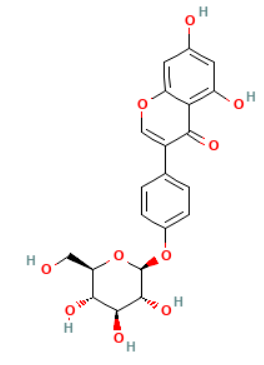 | 5321398 | C1=CC(=CC=C1C2=COC3=CC(=CC(=C3C2=O)O)O)O[C@H]4[C@@H]([C@H]([C@@H]([C@H](O4)CO)O)O)O |
| 13 | Sophora japonica L. | Genistein | Flavonoids | Isoflavones | 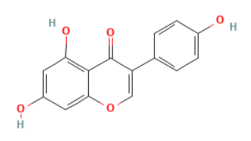 | 5280961 | C1=CC(=CC=C1C2=COC3=CC(=CC(=C3C2=O)O)O)O |
| 14 | Astragalus membranaceus (Fisch.) Bge. | Calycosin | Flavonoids | Isoflavones | 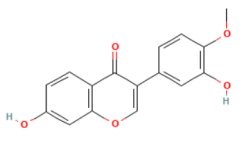 | 5280448 | COC1=C(C=C(C=C1)C2=COC3=C(C2=O)C=CC(=C3)O)O |
| 15 | Astragalus membranaceus (Fisch.) Bge. | Astragalus polysaccharide | Polysaccharides | Polysaccharides | None | None | None |
| 16 | Astragalus membranaceus (Fisch.) Bge. | Astragaloside IV | Terpenoids | Saponins | 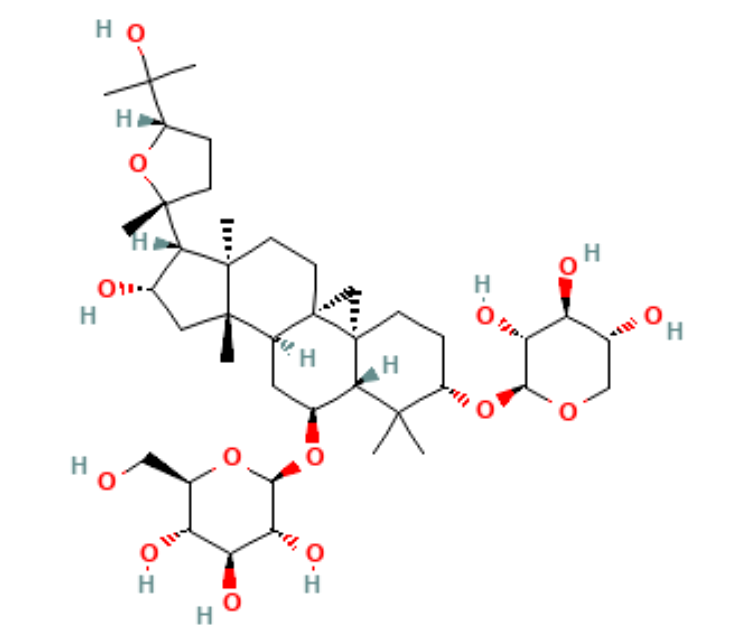 | 13943297 | C[C@]12CC[C@@]34C[C@@]35CC[C@@H](C([C@@H]5[C@H](C[C@H]4[C@@]1(C[C@@H]([C@@H]2[C@]6(CC[C@H](O6)C(C)(C)O)C)O)C)O[C@H]7[C@@H]([C@H]([C@@H]([C@H](O7)CO)O)O)O)(C)C)O[C@H]8[C@@H]([C@H]([C@@H](CO8)O)O)O |
| 17 | Glycine max (L.) Merr. | Daidzein | Flavonoids | Isoflavones | 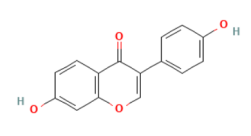 | 5281708 | C1=CC(=CC=C1C2=COC3=C(C2=O)C=CC(=C3)O)O |
| 18 | Glycine max (L.) Merr. | Daidzin | Flavonoids | Isoflavones | 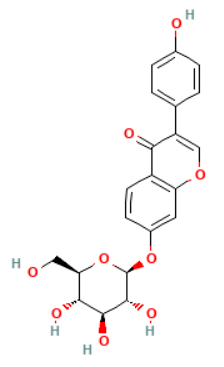 | 107971 | C1=CC(=CC=C1C2=COC3=C(C2=O)C=CC(=C3)O[C@H]4[C@@H]([C@H]([C@@H]([C@H](O4)CO)O)O)O)O |
| 19 | Glycine max (L.) Merr. | Genistin | Flavonoids | Isoflavones | 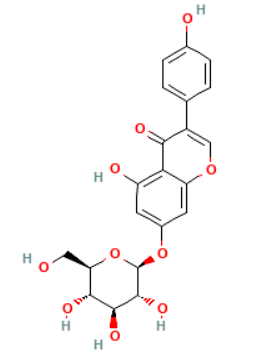 | 5281377 | C1=CC(=CC=C1C2=COC3=CC(=CC(=C3C2=O)O)O[C@H]4[C@@H]([C@H]([C@@H]([C@H](O4)CO)O)O)O)O |
| 20 | Glycine max (L.) Merr. | Glycitin | Flavonoids | Isoflavones | 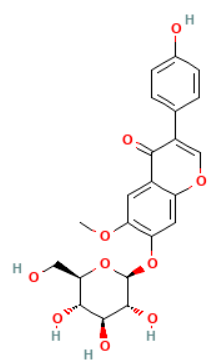 | 187808 | COC1=C(C=C2C(=C1)C(=O)C(=CO2)C3=CC=C(C=C3)O)O[C@H]4[C@@H]([C@H]([C@@H]([C@H](O4)CO)O)O)O |
| 21 | Glycine max (L.) Merr. | Water-soluble soybean fiber | Polysaccharides | Polysaccharides | None | None | None |
| 22 | Glycine max (L.) Merr. | Phytate-removed and deamidated soybean proteins | Proteins & Peptides | Proteins | None | None | None |
| 23 | Pueraria lobata (Willd.) Ohwi | Puerarin | Flavonoids | Isoflavones | 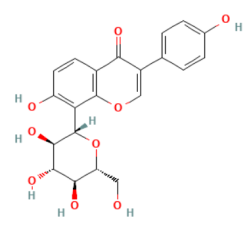 | 5281807 | C1=CC(=CC=C1C2=COC3=C(C2=O)C=CC(=C3[C@H]4[C@@H]([C@H]([C@@H]([C@H](O4)CO)O)O)O)O)O |
| 24 | Lycium barbarum L. | Lycium barbarum polysaccharide | Polysaccharides | Polysaccharides | None | None | None |
| 25 | Nelumbo nucifera Gaertn. | Lotus leaf polysaccharide | Polysaccharides | Polysaccharides | None | None | None |
| 26 | Polygonatum cyrtonema Hua | Polygonatum polysaccharide | Polysaccharides | Polysaccharides | None | None | None |
| 27 | Polygonatum sibiricum Red. | Polygonatum polysaccharide | Polysaccharides | Polysaccharides | None | None | None |
| 28 | Polygonatum sibiricum Red. | Polygonatum sibiricum polysaccharide | Polysaccharides | Polysaccharides | None | None | None |
| 29 | Portulaca oleracea L. | Viscozyme-assisted POP active fraction | Polysaccharides | Polysaccharides | None | None | None |
| 30 | Cornus officinalis Sieb. et Zucc. | Total glycosides of Cornus officinalis | Terpenoids | Iridoid glycosides | None | None | None |
| 31 | Crocus sativus L. | Safranal | Terpenoids | Monoterpene aldehydes | 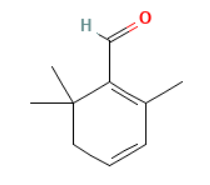 | 61041 | CC1=C(C(CC=C1)(C)C)C=O |
| 32 | Zingiber officinale Rosc. | Cedrol | Terpenoids | Sesquiterpene alcohol | 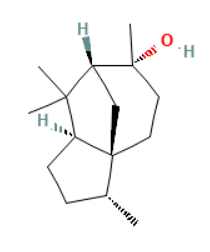 | 65575 | C[C@@H]1CC[C@@H]2[C@]13CC[C@@]([C@H](C3)C2(C)C)(C)O |
| 33 | Ginkgo biloba L. | Bilobalide | Terpenoids | Terpene lactones | 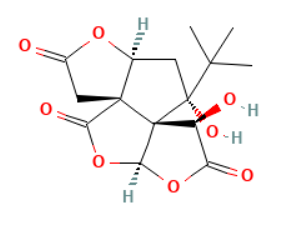 | 73581 | CC(C)(C)[C@@]1(C[C@H]2[C@@]3([C@]14[C@H](C(=O)O[C@H]4OC3=O)O)CC(=O)O2)O |
| 34 | Ginkgo biloba L. | Ginkgolide B | Terpenoids | Terpene lactones | 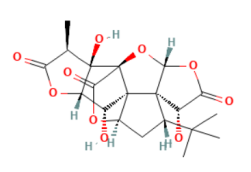 | 11973122 | C[C@@H]1C(=O)O[C@@H]2[C@]1([C@@]34C(=O)O[C@H]5[C@]3([C@@H]2O)[C@@]6([C@@H](C5)C(C)(C)C)[C@H](C(=O)O[C@H]6O4)O)O |
| 35 | Gallus gallus domesticus Brisson | Collagen | Proteins & Peptides | Collagens | None | None | None |
| 36 | Dioscorea opposita Thunb. | HKUOT-S2 protein | Proteins & Peptides | Proteins | None | None | None |
| 37 | Cannabis sativa L. | Cannabidiol | Proteins & Peptides | Cannabinoids | 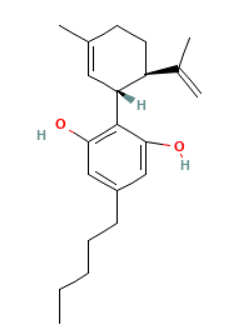 | 644019 | CCCCCC1=CC(=C(C(=C1)O)[C@@H]2C=C(CC[C@H]2C(=C)C)C)O |
| 38 | Codonopsis pilosula (Franch.) Nannf. | Lobetyolin | Other specialized metabolites | Polyacetylenes | 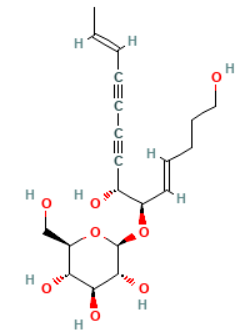 | 14655097 | C/C=C/C#CC#C[C@H]([C@@H](/C=C/CCCO)O[C@H]1[C@@H]([C@H]([C@@H]([C@H](O1)CO)O)O)O)O |
